# Characterization of two melanoma cell lines resistant to BRAF/MEK inhibitors (vemurafenib and cobimetinib)

**DOI:** 10.1186/s12964-024-01788-3

**Published:** 2024-08-23

**Authors:** Magdalena Kot, Aleksandra Simiczyjew, Justyna Wądzyńska, Marcin Ziętek, Rafał Matkowski, Dorota Nowak

**Affiliations:** 1 Department of Cell Pathology, Faculty of Biotechnology, University of Wroclaw, Joliot-Curie 14a, Wroclaw, 50-383 Poland; 2 Department of Oncology, Division of Surgical Oncology, Wroclaw Medical University, Plac Hirszfelda 12, Wroclaw, 53-413 Poland; 3 Lower Silesian Oncology, Pulmonology, and Hematology Center, Plac Hirszfelda 12, Wroclaw, 53-413 Poland

**Keywords:** Melanoma, Cobimetinib, Vemurafenib, Drug resistance, BRAFi/MEKi, Targeted therapy

## Abstract

**Background:**

BRAF (v-raf murine sarcoma viral oncogene homolog B1)/MEK (mitogen-activated protein kinase kinase) inhibitors are used for melanoma treatment. Unfortunately, patients treated with this combined therapy develop resistance to treatment quite quickly, but the mechanisms underlying this phenomenon are not yet fully understood. Here, we report and characterize two melanoma cell lines (WM9 and Hs294T) resistant to BRAF (vemurafenib) and MEK (cobimetinib) inhibitors.

**Methods:**

Cell viability was assessed via the XTT test. The level of selected proteins as well as activation of signaling pathways were evaluated using Western blotting. The expression of the chosen genes was assessed by RT-PCR. The distribution of cell cycle phases was analyzed by flow cytometry, and confocal microscopy was used to take photos of spheroids. The composition of cytokines secreted by cells was determined using a human cytokine array.

**Results:**

The resistant cells had increased survival and activation of ERK kinase in the presence of BRAF/MEK inhibitors. The IC_50_ values for these cells were over 1000 times higher than for controls. Resistant cells also exhibited elevated activation of AKT, p38, and JNK signaling pathways with increased expression of EGFR, ErbB2, MET, and PDGFRβ receptors as well as reduced expression of ErbB3 receptor. Furthermore, these cells demonstrated increased expression of genes encoding proteins involved in drug transport and metabolism. Resistant cells also exhibited features of epithelial-mesenchymal transition and cancer stem cells as well as reduced proliferation rate and elevated cytokine secretion.

**Conclusions:**

In summary, this work describes BRAF/MEK-inhibitor-resistant melanoma cells, allowing for better understanding the underlying mechanisms of resistance. The results may thus contribute to the development of new, more effective therapeutic strategies.

**Supplementary Information:**

The online version contains supplementary material available at 10.1186/s12964-024-01788-3.

## Introduction

Malignant melanoma is a rare [1, 2], but dangerous and invasive skin cancer with a high mortality rate [3, 4]. One of the main reasons for the high aggressiveness of melanoma is the occurrence of a mutated form of serine-threonine kinase BRAF, a component of the mitogen-activated protein kinase (MAPK) signaling pathway, which is detected in about 50% of patients suffering from cutaneous melanoma [5]. The *BRAF V600E* mutation is the most frequent and accounts for 90% of this group [6]. This valine to glutamic acid substitution results in 500-fold increase in kinase activity, leading to increased melanoma progression, proliferation and inhibition of apoptosis [4, 7].

BRAF and MEK inhibitors, as well as immunotherapy, are promising in treating patients with advanced and unresectable malignant melanoma. The therapeutic benefit for patients undergoing combined therapy versus monotherapy is the prolongation of the time of melanoma drug resistance development [8]. The problem of drug resistance has not yet been fully understood, but there are several proposed mechanisms accompanying this phenomenon, such as alternative splicing or amplification *of BRAF V600E*, *MEK* or *NRAS* (*N-ras proto-oncogene)* mutations, and reactivation of MAPK pathway. Additionally, different pathways responsible for cell survival are activated, there is upregulation of receptor tyrosine kinases [9, 10], growth factors activation, metabolic reprogramming [9], changes in the cell interactions with the tumor microenvironment [11], the presence of cancer stem cells (CSCs) [12] and the epithelial–mesenchymal transition (EMT) [13, 14].

Although recently the topic of drug resistance has been intensively studied, there are still many unresolved issues. Therefore in the previous research, we have obtained and characterized two melanoma cell lines resistant to the BRAF inhibitor vemurafenib [15]. However, due to rapidly emerging resistance to monotherapy based on BRAF inhibitors and the reactivation of the MAPK signaling pathway, the current standard treatment for melanoma is the use of a combined therapy based on, i.e., both MEK and BRAF inhibitors. This novel therapeutic approach showed a better clinical response relative to treatment only with BRAF inhibitors [16].

Although the subject of cells resistant to BRAF inhibitors is well studied, our knowledge regarding cells resistant to combined therapy against BRAF and MEK kinases is limited. Therefore, this study was designed to address the problem of drug resistance that also appears in the case of combined therapy. We developed and characterized metastatic melanoma cell lines resistant to BRAF and MEK inhibitors (vemurafenib and cobimetinib, respectively). The proposed inhibitor combination was chosen because it is clinically used in the treatment of melanoma patients with a BRAF mutation [17]. Here, BRAFi/MEKi (BRAF inhibitor/MEK inhibitor)-resistant melanoma cell lines were extensively characterized. We observed differences in biology of melanoma cells resistant to monotherapy compared to mixture of BRAF/MEK inhibitors. The results can better explain resistance to combined therapy and might help chart a better clinical response in patients with advanced malignant melanoma.

## Materials and methods

### Acquisition of resistant melanoma cell lines

Two primary (WM1341D and A375) and two metastatic melanoma cell lines were used to obtain the resistant lines: WM9 (Rockland Immunochemicals, Inc, Royersford, Pennsylvania, USA, purchased in 2018) and Hs294T (ATCC (American Type Culture Collection), Manassas, Virginia, USA, purchased in 2019). The development of resistant cell lines was successful only in the case of cell lines derived from the metastasis: WM9 and Hs294T. Both of them have BRAF V600E mutation [18, 19]. Melanoma cells were cultured in Dulbecco-s Modified Eagle Medium (DMEM; IITD PAN, Wrocław, Poland) containing 1.5 g/l NaHCO3 and 4.5 g/l glucose. The medium was additionally supplemented with antibiotic-antimycotic solution (10 mg/ml streptomycin, 10.000 U/ml penicillin, 25 µg/ml amphotericin B; Thermo Fisher, Waltham, Massachusetts, USA), 2 mM glutamine (Thermo Fisher, Waltham, Massachusetts, USA) and 10% fetal bovine serum (FBS; Thermo Fisher, Waltham, Massachusetts, USA). Cells were cultured in 25 cm^2^ tissue culture flasks (VWR, Radnor, Pennsylvania, USA) and passaged twice a week using 0.25% trypsin/0.05% EDTA (ethylenediaminetetraacetic acid) solution (IITD PAN, Wrocław, Poland). In addition, optimal conditions for the growth of these cells were provided such as 37 °C and 5% CO_2_/95% humidified air.

Resistant melanoma cell lines were obtained by culturing in increasing concentrations of vemurafenib (BRAF kinase inhibitor; Santa Cruz Biotechnology, Dallas, Texas, USA) and cobimetinib (MEK kinase inhibitor, Selleck Chemicals LLC, Houston, Texas, USA). The initial drug concentrations used were 0.05 µM for each inhibitor, and their final concentrations for the obtained resistant melanoma WM9 and Hs294T lines were 0.4 µM vemurafenib and 0.4 µM cobimetinib. Inhibitor concentrations were doubled every two weeks, and the cells were passaged once a week when they reached confluence. In parallel, the control WM9 and Hs294T cells were treated with increasing concentrations of dimethyl sulfoxide (DMSO; AppliChem GmbH, Darmstadt, Germany) because it is the solvent for the inhibitors.

After acquisition of resistance, cells were maintained in a culture medium DMEM with all supplements described above and with the addition of 0.4 µM vemurafenib and 0.4 µM cobimetinib (in the case of control cells DMSO instead of inhibitors was used). Authentication of the derived resistant melanoma cell lines was performed by ATCC using the short tandem repeat (STR) profiling method in 2023. All cell lines were regularly tested for the presence of mycoplasma contamination.

### Proliferation and cytotoxicity assay

A Cell Proliferation Kit II (XTT (2,3-bis-(2-methoxy-4-nitro-5-sulfophenyl)-2 H-tetrazolium-5-carboxanilide); Roche, Basel, Switzerland) was used to measure the cell viability according to the manufacturer’s guidance. Briefly, 5,000 cells were seeded per well on 96-well plates (VWR, Radnor, Pennsylvania, USA) and then the cells were grown for 24 h. To determine the proliferation rate, after changing the medium to the fresh one, XTT was added to all of the investigated samples at time 0 (T0), after 24 h (T24), and 48 h (T48) of cells’ growth. After the XTT mixture addition, cells were incubated for 3 h at 37 °C in 5%CO_2_/95% humidified air. The absorbance at 450 nm was then measured by a µQuant microplate spectrophotometer (Bio-Tek Instruments, Inc., Winooski, VT, USA) using Gen5 software (ver. 2.05, Bio-Tek Instruments, Inc., Winooski, VT, USA). The resulting values were then background corrected. The proliferation rate of the tested cells was calculated by dividing T24 or T48 by T0. Control cells’ proliferation was assumed to be 100%. Each condition was performed in triplicate, and all experiments were conducted at least three times.

To evaluate viability, the cell medium was replaced with one containing DMSO (control condition) or selected concentrations of vemurafenib and cobimetinib (0.0005 µM to 15 µM of each inhibitor) 24 h after seeding. After another 48 h, the fresh medium and XTT mixture were added. Next, cells were incubated for 3 h, and the absorbance was then measured as described above. IC_50_ values were defined as the concentration of the inhibitors at which the drugs exert half of their maximal inhibitory effect. IC_50_ values were calculated using GraphPad Prism 7 software after examining the viability of resistant and control WM9 and Hs294T melanoma cell lines treated with a range of concentrations of tested inhibitors.

### Synchronization of melanoma cell growth for cell cycle analysis

Due to the significant differences in the proliferation rate of control and resistant melanoma cells, synchronization of the cells was performed before cell cycle analysis. Cells were seeded and cultured under standard conditions, and the medium was changed to a medium without FBS after reaching approximately 30% confluence and cultured for 48 h. After this, FBS was re-added to the culture medium for another 24 h. Cells were then collected for the cell cycle phase distribution analysis as described in point 4. The cells collected for this analysis did not exceed approximately 50% confluence to avoid the influence of contact inhibition on the interpretation of this experiment data through the growth suppression.

### Cell cycle phases distribution analysis

Synchronized cells were washed with Ca^2+/^Mg^2+^-free PBS, trypsinized, centrifuged (100 x g, 5 min, RT) fixed with ice-cold 70% ethanol, and then incubated for at least 24 h in − 20 °C. Cells were then washed twice with PBS, centrifuged (1000 x g, 5 min, RT), incubated with RNase A (8 µg/ml, 45 min, RT), and stained with propidium iodide (1 mg/ml, 30 min, RT). Next samples were transferred on ice and subsequently analyzed with a NovoCyte flow cytometer (ACEA) and ACEA NovoExpress software (ver. 1.2.4, ACEA Biosciences). At least 10,000 cells gated for singlets were acquired for each sample. At least three independent experiments were performed for each cell line.

### Acquisition of conditioned medium from melanoma cells

Media used for the examination of secreted protein levels were obtained as we described previously [20]. Briefly, resistant and control melanoma cells were cultured in tissue culture flasks until they reached about 70–80% confluence. Then, cells were washed three times with PBS and then fresh medium without FBS was added for 72 h. After that, media were collected, centrifuged for 15 min at 1000 x g, and concentrated with the use of Amicon^®^ Ultra-4 Centrifugal Filters (Merck Millipore, Burlington, Massachusetts, USA) according to the manufacturer’s instructions.

### Spheroids acquisition with the use of the hanging drop technique

To obtain spheroids, control, and resistant melanoma cells were trypsinized and counted. Then, 5000 cells were spotted on the cover of the culture dish to obtain a hanging drop. The final volume of one drop was 30 µl. PBS was placed at the bottom of the dish to prevent the drops from drying out. The spheroids were cultured for two weeks with a change of medium approximately every three days. Cells were then stained and observed under a confocal microscope.

### Cytochemical staining of spheroids

For staining, spheroids from the cover of the culture dish were rinsed in 1 ml PBS and then centrifuged for 5 min at 2300 x g at room temperature. Spheroids from the bottom of the Eppendorf tube were resuspended in 200 µl of 4% formaldehyde and incubated for 15 min in the refrigerator. They were then centrifuged again under the same conditions. After that the spheroids were resuspended in 200 µl of PBS and centrifuged again as before. The spheroids were then resuspended in 200 µl of 0.5% Triton in PBS and incubated for 30 min at room temperature with shaking. In the next step cells were resuspended in 0.5% Triton in PBS and centrifuged as before. The spheroids were then blocked with a 1% BSA (bovine serum albumin) in 0.1% Triton in PBS solution for 1 h at room temperature with shaking and then centrifuged as above. Phalloidin-Alexa Fluor 568 (Thermo Fisher, Waltham, Massachusetts, USA) was diluted 100 times in 1% BSA in 0.1% Triton in PBS and incubated with the spheroids overnight at 4 °C with shaking. The next day, the spheroids were again washed in 0.5% Triton in PBS and centrifuged as above. The pellet was then resuspended in 50 µl of PBS and applied to a slide. Excess PBS was removed, and DAKO mounting solution was added followed by a coverslip.

### Western blotting analysis

To collect the lysates, resistant and control melanoma WM9 and Hs294T cells were seeded in appropriate culture dishes until they reached confluence. The cells were then transferred on ice and washed three times with PBS. Cell lysates were then collected in urea buffer (50 mM Tris, pH 7.4, 74 mM urea, 1 mM dichlorodiphenyltrichloroethane, 8.6% sucrose, 5% sodium dodecyl sulfate (SDS) with the addition of phosphatase and protease inhibitors cocktails (Sigma Aldrich, Burlington, Massachusetts, USA)). The concentration of proteins was determined via a standard bicinchoninic acid (BCA) procedure (Thermo Fisher, Waltham, Massachusetts, USA). Samples containing the same amount of protein (10 µg for cell lysates and 5 µg for conditioned media) were separated by 10% polyacrylamide gel electrophoresis with the addition of sodium dodecyl sulfate (SDS-PAGE) according to Laemmli reports [21] and then transferred to nitrocellulose membranes as described by Towbin et al. [22].

Primary antibodies were directed against pERK1/2 (phosphorylated extracellular signal-regulated kinases 1/2) (Cell Signaling Technologies, #9101, 1:1000), AKT (Protein kinase B) (Cell Signaling Technologies, #4691, 1:1000), pAKT (phosphorylated protein kinase B) (Cell Signaling Technologies, #9271, 1:1000), p38 (p38 mitogen-activated protein kinase) (Cell Signaling Technologies, #8690, 1:1000), p-p38 (phosphorylated p38 mitogen-activated protein kinase) (Cell Signaling Technologies, #4511, 1:1000), JNK (c-Jun N-terminal kinase) (Santa Cruz Biotechnology, sc-7345, 1:200), p-JNK (phosphorylated c-Jun N-terminal kinase) (Santa Cruz Biotechnology, sc-6254, 1:200), CYP1A1 (Cytochrome P450 family 1 subfamily A member 1) (Santa Cruz Biotechnology, sc-25304, 1:200), ALCAM (CD166 antigen) (Santa Cruz Biotechnology, sc-74558, 1:200), TGFβRIII (Transforming growth factor-beta receptor III) (Cell Signaling Technologies, #2519, 1:1000), TGFβRI (Transforming growth factor-beta receptor I) (Santa Cruz Biotechnology, sc-398, 1:200), SOX2 (SRY-box transcription factor 2) (Cell Signaling Technologies, #3578, 1:1000), CDK6 (cyclin-dependent kinase 6) (Cell Signaling Technologies, #3136, 1:1000), p18 (CDKN2C (cyclin dependent kinase inhibitor 2 C) (Cell Signaling Technologies, #2896, 1:1000), p21 ((CDKN1A) cyclin dependent kinase inhibitor 1 A) (Cell Signaling Technologies, #2947, 1:1000), and p27 ((CDKN1B) cyclin dependent kinase inhibitor 1B) (Cell Signaling Technologies, #3686, 1:1000). Secondary antibodies (anti-mouse and anti-rabbit) conjugated with horseradish peroxidase (Cell Signaling Technologies, 7076 and 7074 respectively, 1:4000) were used according to the manufacturers’ instructions.

Immunoblots were developed using Clarity Western ECL Substrate (Bio-Rad, Hercules, California, USA) or Clarity Max Western ECL Substrate (Bio-Rad, Hercules, California, USA) and then scanned with the use of ChemiDoc (Bio-Rad, Hercules, California, USA). Bands’ signal intensities were transformed to the numerical values using ImageLab Software (v. 6.0, Bio-Rad, Hercules, California, USA) and normalized to the total protein content obtained by Ponceau S staining. The results were entered into GraphPad Prism7 software, and statistical analysis was performed and charts were prepared. At least three independent experiments from different biological repetitions were performed.

### Real-time PCR analysis

To explore the level of selected genes, RNA was isolated using a miRNeasy Mini Kit (Qiagen, Hilden, Germany) followed by DNase I digestion applying RNase-Free DNase Set (Qiagen, Hilden, Germany) and reverse transcription reaction with the use of a High-Capacity cDNA Reverse Transcription Kit (Applied Biosystems, Waltham, Massachusetts, USA). All steps were done according to the manufacturers’ guidance. Quantitative PCR was performed using PowerUp™ SYBR™ Green Master Mix on a StepOnePlus system (Applied Biosystems, Waltham, Massachusetts, USA). The results were normalized to the reference HPRT1 gene expression. Sequences of primers used are shown in Table 1.

**Table 1:** Sequences of primers used for quantitative PCR analysis

Gene	Forward Primer 5’ – 3’	Reverse Primer 5’ – 3’
NRAS	GAGTACAAACTGGTGGTGGTTGGA	ATTGGTCTCTCATGGCACTGTACT
PDGFRβ	GCTCACACTGACCAACCTCAC	GTCTGTTACTCGGCATGGAATGG
ABCA1	GGATTATCTGTAATGCCAACAA	CTGGATTTCTTGATCTGCTGT
ABCC2	ACCTGCCACTTTGTTTTGAGCA	AGAGTCTTCTGTGAGTACAAGGGC
ABCG2	GCATTTACTGAAGGAGCTGTGTTAAGTT	CTAATAACGAAGATTTGCCTCCACCT
MITF	CTATCAGGTGCAGACCCACCT	GTAAGCATAGCCATGGGGCTG
CD24	TGAAGAACATGTGAGAGGTTTGAC	GAAAACTGAATCTCCATTCCACAA
NANOG	CACCTATGCCTGTGATTTGTGGG	TGGGACTGGTGGAAGAATCAGG
SLUG	CAGCGAACTGGACACACATACAG	GGAGTATCCGGAAAGAGGAGAGAG
SOX10	GTCAACGGCGCCAGCAAAAG	AGGGGCGCTTGTCACTTTCG
IL6	GCCCTGAGAAAGGAGACATG	CAAGTCTCCTCATTGAATCCAGAT
IL1β	ATGGCTTATTACAGTGGCAATG	GTAGTGGTGGTCGGAGATTC

*List of abbreviations*: *ABCA1* (ATP-binding cassette transporter 1), *ABCC2* (multidrug resistance-associated protein 2), *ABCG2* (ATP binding cassette subfamily G member 2), *CD24* (cluster of differentiation 24), *MITF* (microphthalmia-associated transcription factor), *NANOG* (nanog homeobox), *NRAS* (N-ras proto-oncogene), *PDGFRB* (platelet-derived growth factor receptor beta), *SLUG* (SNAI1, snail family transcriptional repressor 2), *SOX10* (SRY-box transcription factor 10), *IL6* (interleukin 6), and *IL1β* (interleukin 1 β)

Specific TaqMan^®^ probes were used to examine the level of expression of genes such as EGFR (ErbB1, epidermal growth factor receptor), MET (MET proto-oncogene, receptor tyrosine kinase), HER2 (ErbB2, erb-b2 receptor tyrosine kinase 2), HER3 (ErbB3, erb-b2 receptor tyrosine kinase 3), and GAPDH (glyceraldehyde-3-phosphate dehydrogenase) (Applied Biosystems, Hs01076091-m1, Hs01565576-m1, Hs01001580, Hs00176538-m1, and Hs02758991-g1, respectively); GAPDH served as a reference. Quantitative PCR was performed with the use of TaqMan^®^ Universal Master Mix II (Applied Biosystems, Waltham, Massachusetts, USA) according to the manufacturer’s instructions.

The results were normalized to appropriate reference genes expression based on the comparative CT (threshold cycle value) method (ΔCT = 2^-(CT gene of interest − CT housekeeping gene). At least three independent experiments were performed—each sample in duplicate.

### Human cytokine array

A Proteome Profiler Human Cytokine Array Kit (R&D systems, Minneapolis, Minnesota, USA) was used to determine the elements of resistant and control WM9 and Hs294T cells’ secretome. In this method, antibodies immobilized on the membrane allow one to identify different chemokines and cytokines that are present in the analyzed samples. The protein concentration of the media derived from investigated cell lines was equalized to 50 µg, and the entire procedure was performed according to the manufacturer’s instruction as described earlier [23]. Briefly, samples were agitated with biotinylated detection antibodies and then applied to nitrocellulose membranes and incubated overnight. The next day, the membranes were washed several times, and the chemiluminescent signal was explored with the use of ChemiDoc Imaging System (Bio-Rad, Hercules, California, USA) and analyzed with ImageLab Software (v. 6.0, Bio-Rad, Hercules, California, USA). After background correction, the results were normalized to the mean of reference dots presented on each membrane. Quantitative analysis of the signal was performed. The results were normalized to reference spots and are shown in the form of a heatmap prepared using GraphPad Prism7 software.

### Statistical analysis

All data are given as the means ± standard deviation (SD), and their significance was evaluated with GraphPad Prism 7 software using a one-way ANOVA followed by Tukey’s test or Welch’s t-test.

## Results

### Obtaining BRAFi/MEKi-treatment-resistant WM9 and Hs294T melanoma cell lines

Resistant melanoma cell lines were obtained as described in the ‘Materials and Methods’ section. Briefly, WM9 and Hs294T melanoma cells were cultured in increasing concentrations of BRAF and MEK inhibitors (vemurafenib and cobimetinib, respectively) starting at 0.05 µM for both drugs. The final inhibitor concentrations constituted 0.4 µM vemurafenib and 0.4 µM cobimetinib. The control cells (CTRL) were WM9 and Hs294T cells treated with regular media with DMSO at the concentration used for drug delivery.

**Fig. 1:**
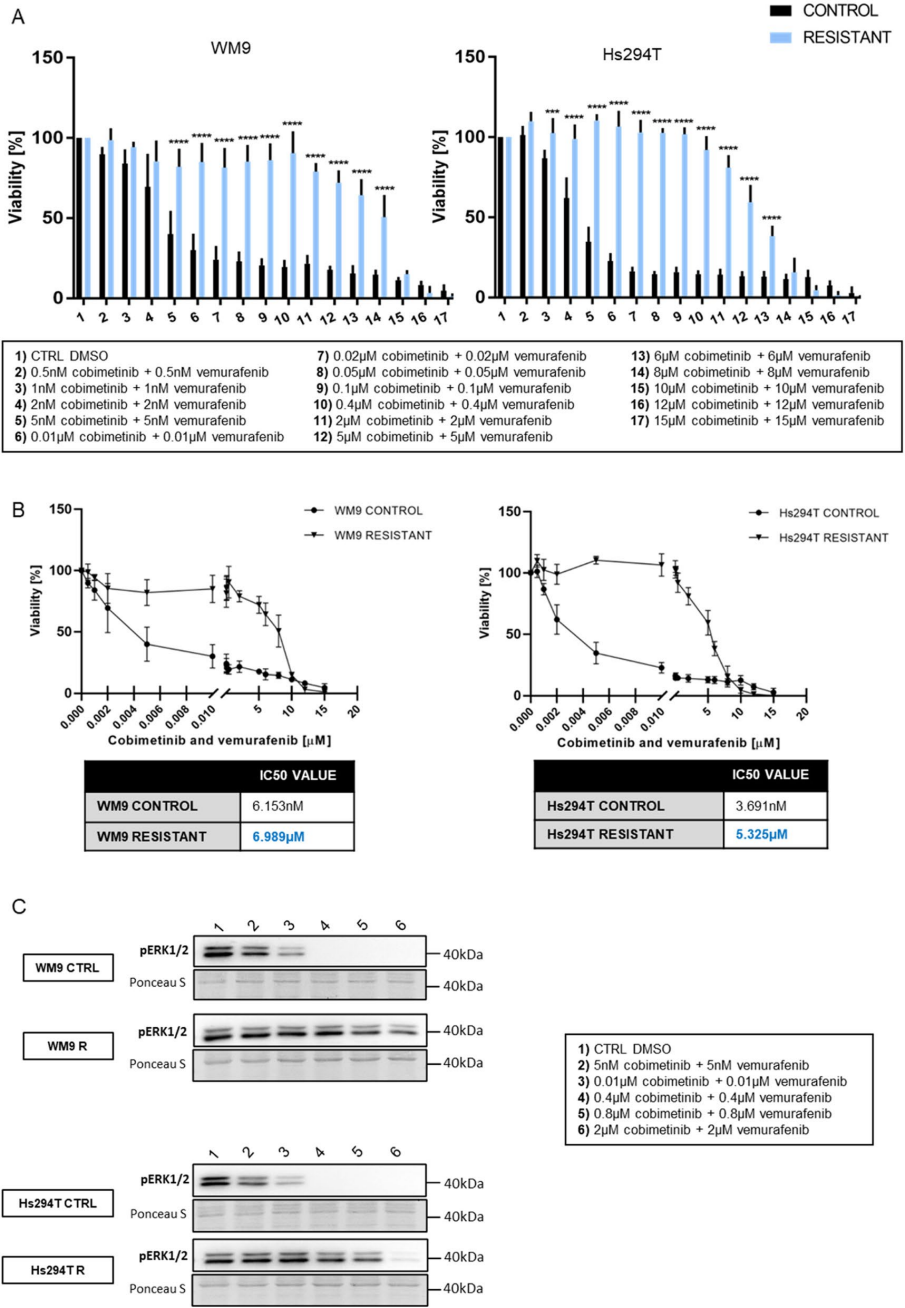
Sensitivity of control and resistant melanoma cell lines to the vemurafenib and cobimetinib. **A** The viability of the control and resistant melanoma cells after 48 h of treatment with BRAF/MEK inhibitors was measured using the XTT assay. **B** Based on the viability measurement and the XTT test, IC_50_ values were determined for control and resistant cell lines and then calculated using GraphPad Prism 7 program. **C** The level of phosphorylated ERK1/2 kinase after 24 h of incubation with BRAF/MEK inhibitors in control (CTRL) and resistant (R) melanoma cell lines was verified by Western Blotting in cell lysates. Control (CTRL) constitutes WM9 and Hs294T cells treated with regular media with DMSO at the concentration used for drug delivery. Representative blotting membranes of three independent experiments are shown. The loading control was the total protein content assessed by Ponceau S staining. The graphs show the average values from at least three independent experiments ± SD. Asterisks in the graphs indicate statistical significance (p) at the level of *** ≤ 0.001, **** ≤ 0.0001

To confirm the resistance acquisition by melanoma cells, a viability test was performed in which resistant and control cells were exposed to increasing concentrations of BRAF/MEK inhibitors. The analysis showed significantly reduced sensitivity of both resistant melanoma cell lines to the BRAFi/MEKi combination. Control cells exhibited much lower viability than resistant cells at the same drug concentrations (Fig. 1A). The IC_50_ values were more than 1000 times higher in resistant cells versus control ones (6,153 nM for control and 6,989 µM for resistant WM9 cell line, and 3,691 nM for control and 5,325 µM for resistant Hs294T cell line, Fig. 1B). Additionally, the phosphorylation of ERK1/2, which is a direct downstream effector of BRAF, was inhibited in control cells treated with the drugs, while it was still observed under the same conditions in resistant cells (Fig. 1C).

### Increased activation of signaling pathways in resistant melanoma cell lines

**Fig. 2:**
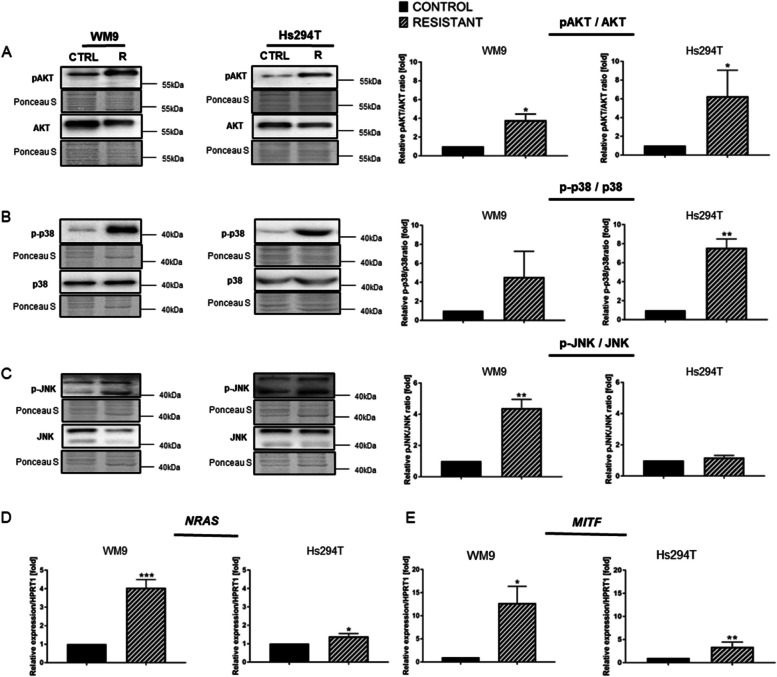
Activation of signaling pathways in resistant and control melanoma cell lines. The level of total or phosphorylated (**A**) AKT, (**B**) p38, and (**C**) JNK in cell lysates was determined using Western blotting analysis. The signal was normalized to the total protein content assessed by Ponceau S staining. Representative blotting membranes of at least three biological repetitions are shown. The expression level of the (**D**) *NRAS* and (**E) ***MITF* genes was estimated by real-time PCR and with the use of designed primers. *HPRT1* constituted a reference gene. Control (CTRL) constitutes WM9 and Hs294T cells treated with regular media with DMSO at the concentration used for drug delivery. The graphs show average values from at least three independent experiments ± SD. Asterisks indicate statistically important differences between tested and control cells. The significance level was set at *p* ≤ 0.05 (*), *p* ≤ 0.01 (**), and *p* ≤ 0.001 (***)

BRAFi/MEKi resistance acquisition can be connected with increased activation of signaling pathways, and thus the level of activation of elements of PI3K/AKT (phosphoinositide 3-kinase/protein kinase B) and MAPK pathways was verified. The results show a statistically significant elevation of the pAKT/AKT ratio in both resistant melanoma cell lines versus control lines (Fig. 2A). Two other proteins were also examined: p38 and JNK, which apart from ERK1/2 are the main components of the MAPK pathway. Western Blotting analysis facilitated observations of an increased p-p38/p38 ratio (statistically significant in case of Hs294T R cell line) as well as elevated p-JNK/JNK ratio (statistically significant in case of WM9 R cell line) in resistant melanoma cell lines versus control cells (Fig. 2B, C). Due to reports indicating the role of NRAS in resistance to vemurafenib, its level was measured in the BRAFi/MEKi-resistant melanoma cell lines. Indeed, an increase in the expression of gene encoding *NRAS* was observed in both resistant melanoma cell lines; this increase was 4-fold higher in WM9 R (Fig. 2D). Moreover, we verified the level of another protein, key during melanoma progression - microphthalmia-associated transcription factor (MITF). Interestingly, its amount was increased in resistant cells compared to sensitive ones (Fig. 2E).

### Changes in expression of genes encoding selected tyrosine kinase receptors occurring in resistant melanoma cell lines

**Fig. 3:**
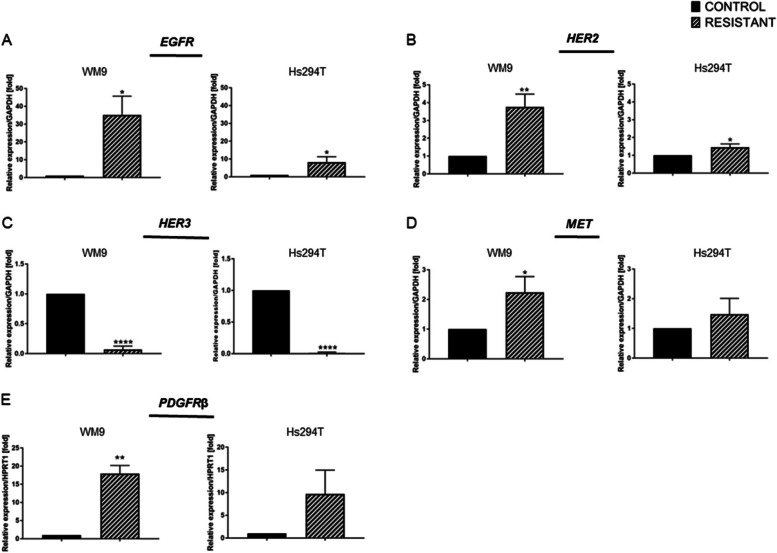
Expression of selected tyrosine kinase receptors in resistant melanoma cell lines. **A*** EGFR*, (**B**)* HER2*, (**C**) *HER3*, (**D**)* MET*, and (**E**)* PDGFRβ* receptor expression was assessed in control and resistant melanoma WM9 and Hs294T cells using real-time PCR. Taqman probes were used to evaluate the expression level of *EGFR*, *HER2*,* HER3*, and *MET* receptors, and *GAPDH* served as a housekeeping gene. The expression level of the *PDGFRβ* receptor was determined by utilizing the designed primers. *HPRT1* constituted a reference gene. Control (CTRL) constitutes WM9 and Hs294T cells treated with regular media with DMSO at the concentration used for drug delivery. The graphs show average values from at least three independent experiments ± SD. The asterisks indicate the significance level at *p* ≤ 0.05 (*), *p* ≤ 0.01 (**) and *p* ≤ 0.0001 (****)

According to many reports indicating that drug resistance is often accompanied by overexpression of receptor tyrosine kinases (RTKs), the expression levels of *EGFR*, *HER2*,* HER3*, *MET*, and *PDGFRβ* receptors were verified in both resistant melanoma cell lines. Real-time PCR analysis verified the expression of three receptor tyrosine kinases from the HER family: *EGFR* (also known as *HER1 or ErbB1*), *HER2* (also named *ErbB2)*, and *HER3* (the same as *ErbB3)*. EGFR and HER2 in both resistant melanoma cell lines were significantly elevated on mRNA level (Fig. 3A, B). Surprisingly, the expression of *HER3* in both resistant melanoma lines was significantly reduced (Fig. 3C). Increased expression was also observed in the case of another gene frequently overexpressed in melanoma: hepatocyte growth factor receptor, *MET*. Nevertheless, a statistically significant increase was only noticed in the WM9 R cell line (Fig. 3D). The last tested receptor from the RTK family was platelet-derived growth factor receptor beta (*PDGFRβ*). qPCR analysis showed an increase in *PDGFRβ* mRNA levels in resistant melanoma cell lines with statistical significance in the WM9 R line (Fig. 3E).

### Changes in the expression of proteins involved in drug efflux and metabolism in resistant melanoma cells

**Fig. 4:**
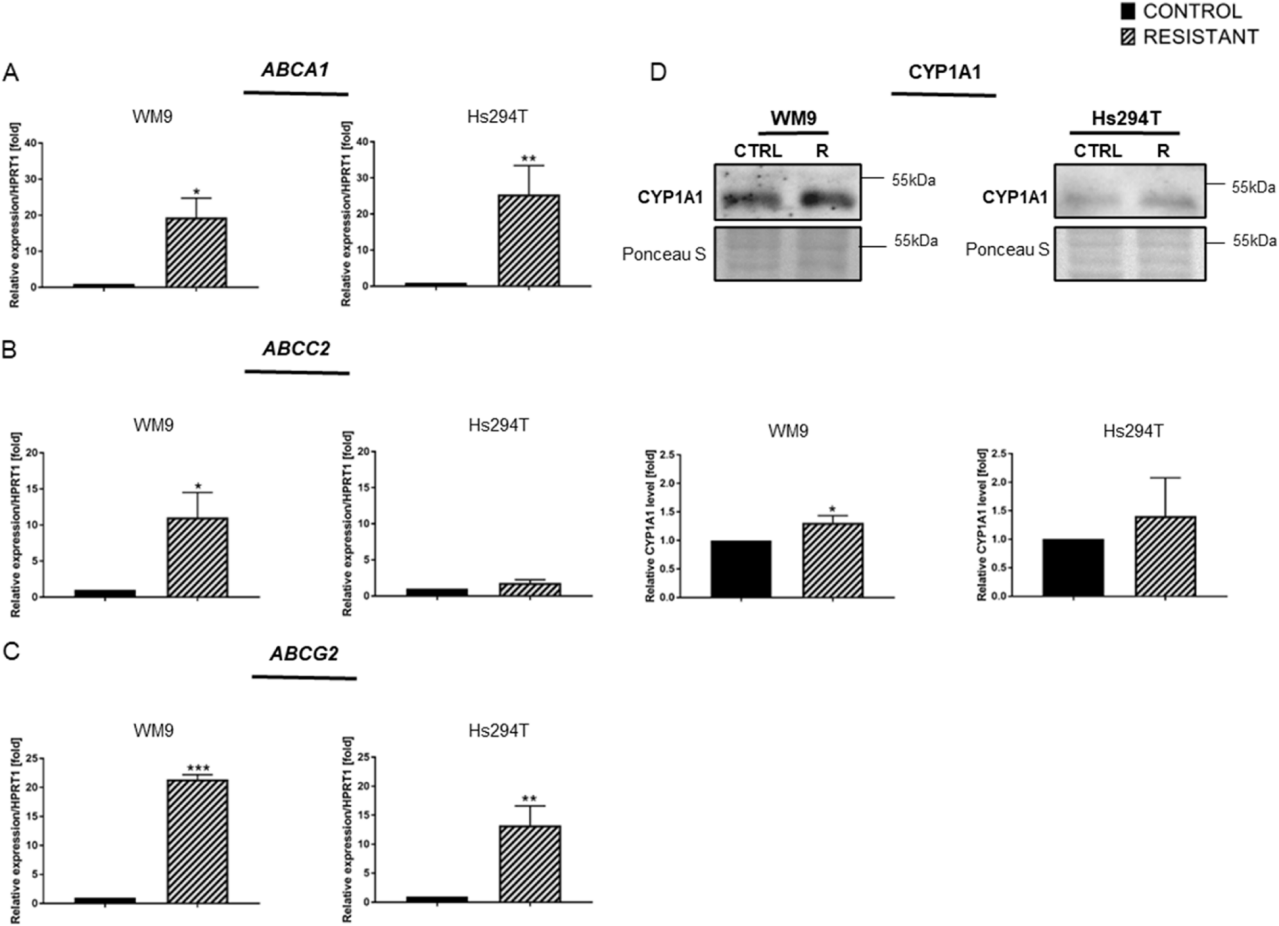
Expression of genes encoding proteins involved in drug transport and metabolism in resistant melanoma cells. **A*** ABCA1*, (**B**) *ABCC2*, and (**C**) *ABCG2* transporter expression in control and resistant melanoma WM9 and Hs294T cell lines. Real-time PCR was performed with *HPRT1* as a reference gene. **D** The level of CYP1A1 protein in cell lysates was determined using Western blotting. The signal was normalized to the total protein content assessed by Ponceau S staining. Representative blotting membranes of three independent experiments are shown. Control (CTRL) constitutes WM9 and Hs294T cells treated with regular media with DMSO at the concentration used for drug delivery. The graphs show average values from at least three independent experiments ± SD. Asterisks in the graphs indicate statistical significance (p) at the level of * ≤ 0.05, ** ≤ 0.01, and *** ≤ 0.001

ATP-binding cassette (ABC) transporters are involved in the efflux of anti-cancer drugs from cells, and thus are associated with the development of drug resistance. We evaluated the expression level of some of the ABC transporters in control and resistant WM9 and Hs294T cell lines. The real-time PCR analysis showed a significant (over 12-fold) increase in *ABCA1* expression in both resistant melanoma cell lines compared to control cells (Fig. 4A). *ABCC2* transporter expression was also elevated on mRNA levels, with a more pronounced increase in WM9 R cell line (Fig. 4B). To the best of our knowledge, this is the first result showing an increase in the expression of the *ABCA1* and *ABCC2* transporters in BRAFi/MEKi-resistant cells. The last member from the ABC transporters family included in this study was ABCG2 — it is involved in the removal of drugs from target cells. As expected, qRT PCR analysis revealed a statistically significant increase in the expression of the *ABCG2* transporter in resistant WM9 and Hs294T cell lines versus control ones (Fig. 4C).

Cytochrome P450 enzymes (CYPs) are another group of proteins that may have a significant impact on the effectiveness of treatment of various types of cancers, including melanoma. A frequently described protein belonging to this family is CYP1A1. Its expression is closely related to the effectiveness of anti-cancer treatment. Western blotting revealed an elevated level of CYP1A1 protein in resistant melanoma cell lines, but this was statistically significant only for WM9 R cell line (Fig. 4D).

### Reduced proliferation rate, changed distribution of cell cycle phases, and decreased level of proteins regulating cell cycle in resistant melanoma cells

**Fig. 5:**
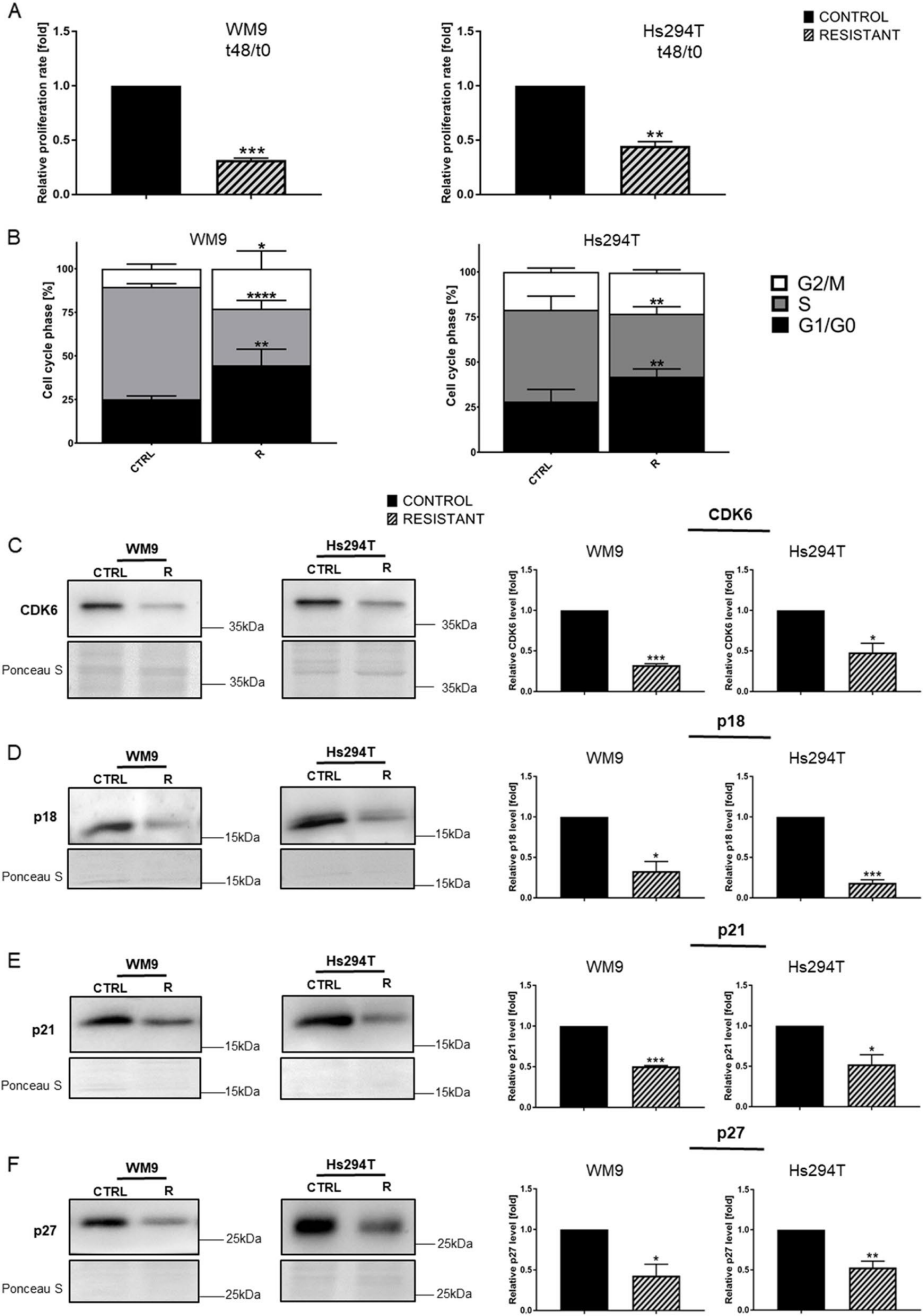
Changes in proliferation rate and cell cycle phases distribution in resistant melanoma cell lines. **A** Melanoma WM9 and Hs294T cells were seeded on a 96-well plate, and their proliferation rate was calculated as a ratio of the spectrophotometric signal after 48 h divided by the signal at t0. **B** Cell cycle analysis in WM9 and Hs294T melanoma control cells and cells resistant to treatment with BRAFi/MEKi. Western blot analysis of the level of proteins involved in cell cycle regulation: (**C**) CDK6, (**D**) p18, (**E**) p21, and (**F**) p27 in cell lysates obtained from control and resistant melanoma cells. The signal was normalized to the total protein content assessed by Ponceau S staining. Representative blotting membranes of three independent experiments are shown. Control (CTRL) constitutes WM9 and Hs294T cells treated with regular media with DMSO at the concentration used for drug delivery. Cells for cell cycle analysis as well as those used for Western blotting analysis were synchronized to obtain reliable results for cells dividing at widely varying rates as described in the ‘Materials and Methods’ section. The graphs show average values from at least three independent experiments ± SD. Asterisks in the graphs indicate statistical significance (p) at the level of * ≤ 0.05, ** ≤ 0.01, *** ≤ 0.001, and **** ≤ 0.0001

We observed a significantly lower rate of division in resistant WM9 and Hs294T cells versus controls. Viability tests were thus performed to quantitatively confirm our observations. The results indicated a statistically significant reduction in the proliferation rate of both BRAFi/MEKi-resistant melanoma cell lines after 48 h of cell culture versus control cells (Fig. 5A). These findings verified whether the emergence of resistance in melanoma cells was accompanied by changes in the distribution of cell cycle phases. The analysis showed an increase in the G1/G0 phase as well as a decrease in the S phase in both resistant melanoma cell lines. There was also an increase in the G2/M phase but only in the case of the WM9 R cell line (Fig. 5B, Supplementary Fig. 1). Some proteins involved in cell cycle regulation were also investigated due to these differences: decreased levels of cyclin-dependent kinase 6 (CDK6) as well as p18, p21, and p27 were observed in both resistant melanoma cell lines versus control cells.

### Resistant melanoma cell lines reveal selected features characteristic of cancer stem cells (CSCs)

**Fig. 6:**
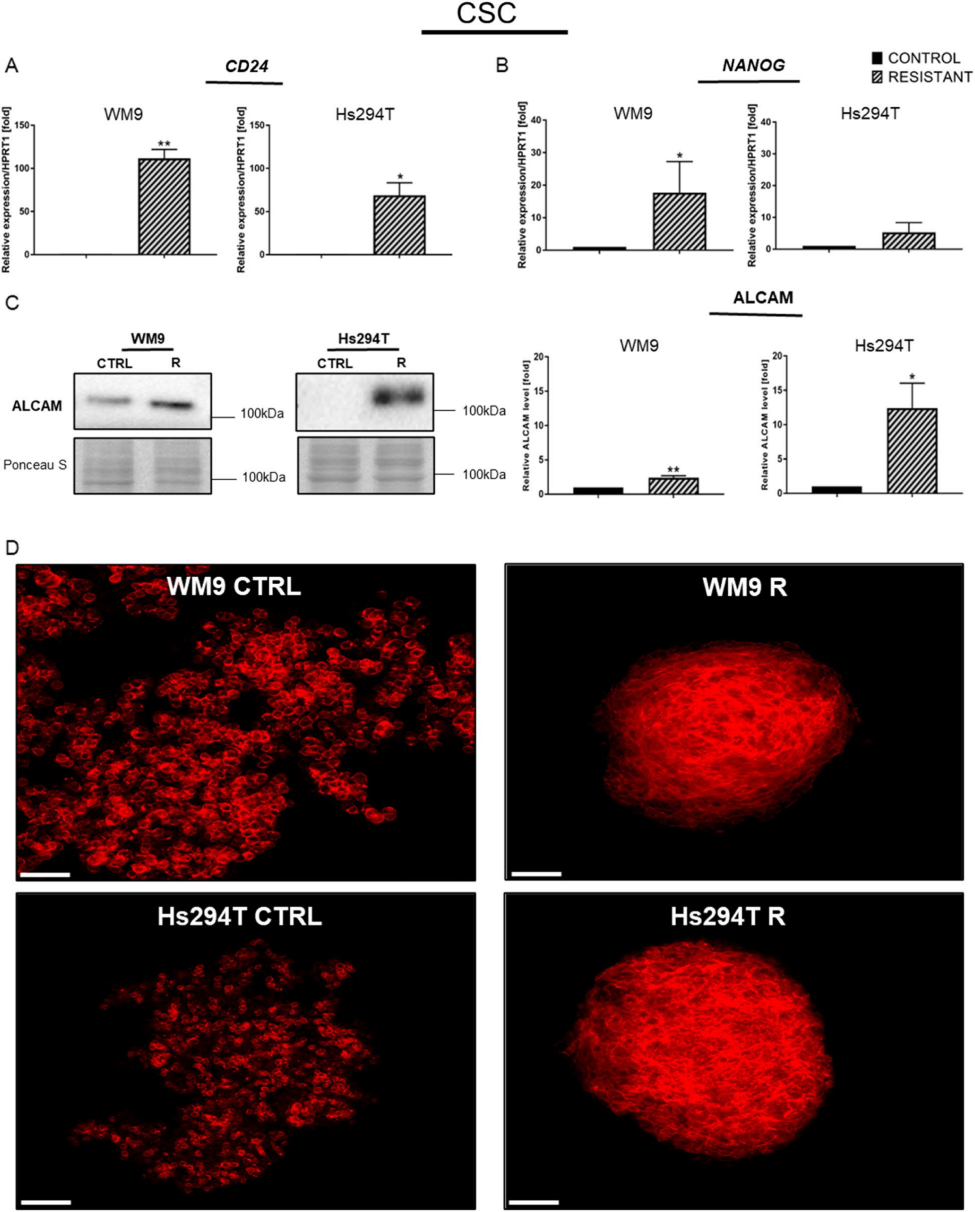
Features characteristic of cancer stem cells (CSC) in resistant and control melanoma cell lines. Elevated expression of CSC markers like (**A**)* CD24* and (**B**) *NANOG* accompanied by (**C**) increased level of another CSC protein, ALCAM. *HPRT1* is a reference gene. The Western blotting signal was normalized to the total protein content assessed by Ponceau S staining. Representative blotting membranes of three independent experiments are shown. The graphs show average values from at least three independent experiments ± SD. Asterisks in the graphs indicate statistical significance (p) at the level of * ≤ 0.05 and ** ≤ 0.01. **D **Spheroids from control and resistant melanoma cells were obtained and then fixed and stained using phalloidin-Alexa Fluor 568 to visualize F-actin (red). Representative images taken with a fluorescence microscope are shown. Scale bar is 100 μm. Control (CTRL) constitutes WM9 and Hs294T cells treated with regular media with DMSO at the concentration used for drug delivery

There is a correlation between cancer stem cells and the emergence of drug resistance [24]. The reduced proliferation rate of resistant melanoma cells is one of the features accompanying the presence of cancer stem cells (CSCs) [25]. Real-time PCR analysis revealed an increase in the mRNA level of genes encoding CSC markers: *CD24* (Fig. 6A) and *NANOG* (its expression was significantly elevated only in WM9 R cells) (Fig. 6B). Increased expression of the gene encoding *CD24* in the WM9 R cell line was approximately 100-fold higher, and over 50-fold higher in the Hs294T R cell line versus control cell lines. Western blotting showed an increase in the level of another CSC marker, ALCAM, in both resistant melanoma cell lines (Fig. 6C).

CSCs can form spheroids [26–28], and we attempted to obtain spheroids from control and resistant melanoma cells. Figure 6D shows that resistant melanoma cells form structures that are more compact, tight, and spheroid-like than control cells, which are less compact and more dispersed.

### Resistant melanoma cells exhibit epithelial-mesenchymal transition features

**Fig. 7:**
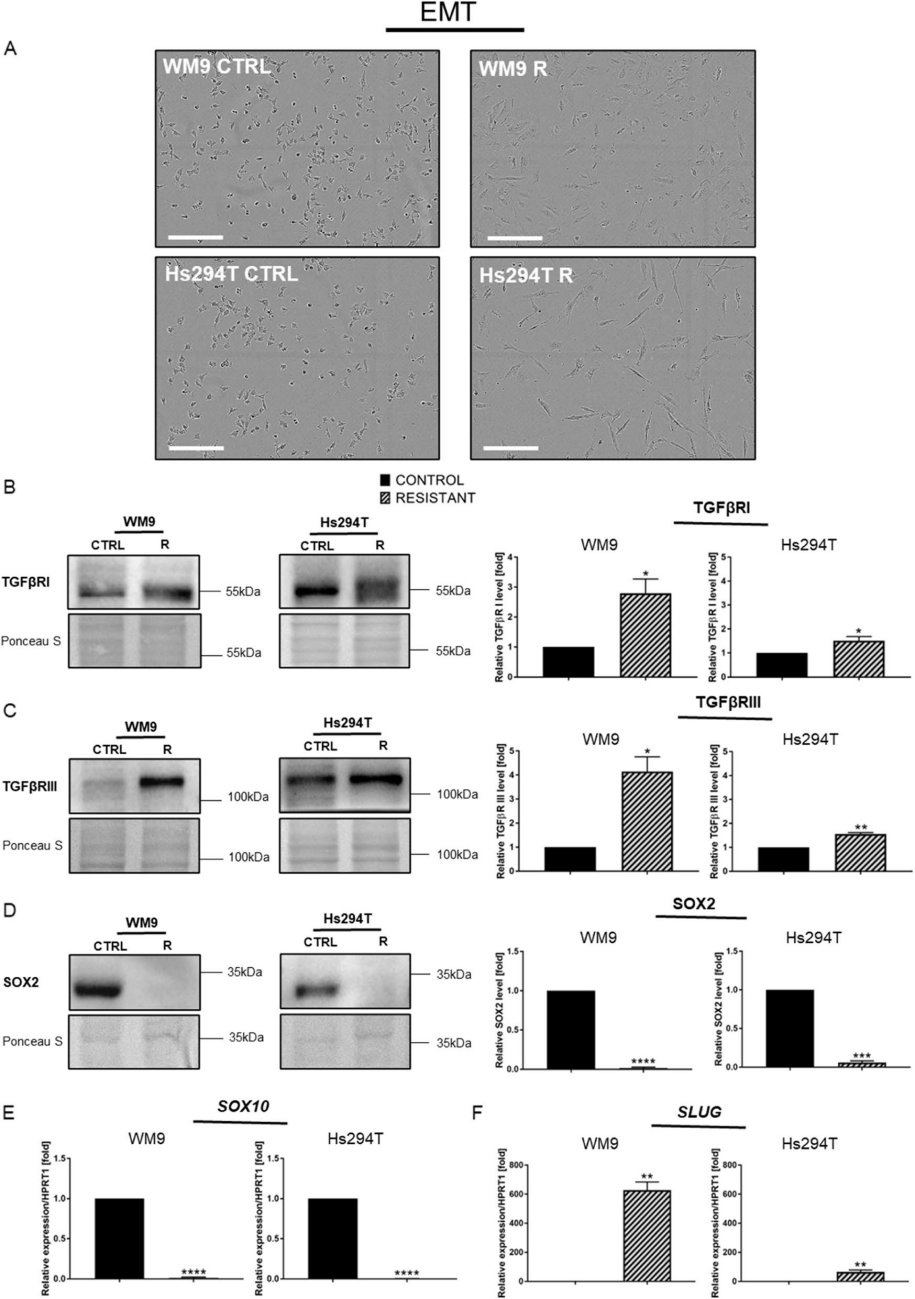
Resistant melanoma cells display spindle-like shape and features characteristic of epithelial-mesenchymal transition (EMT). **A** Photographs revealing the morphology of resistant and control melanoma cells taken with a light microscope. Scale bar 300 μm. Western Blotting analysis of (**B**) TRGβR I, (**C**) TRGβR III and (**D**) SOX2 coupled with expression level analysis of (**E**) *SOX10* and (**F**) *SLUG* genes. *HPRT1* is a reference gene. The Western blotting signal was normalized to the total protein content assessed by Ponceau S staining. Representative blotting membranes of three independent experiments are shown. Control (CTRL) constitutes WM9 and Hs294T cells treated with regular media with DMSO at the concentration used for drug delivery. The graphs show the average values from at least three independent experiments ± SD. Asterisks in the graphs indicate statistical significance (p) at the level of * ≤ 0.05, ** ≤ 0.01, *** ≤ 0.001 and **** ≤ 0.0001

The derived resistant melanoma cell lines exhibited an elongated and spindle-like shape. This is a feature typical of cells that undergo EMT [29] (Fig. 7A). EMT is a process closely related to CSC state [26]. At the protein level, the expression of EMT markers TGFβRI and TGFβRIII was increased (Fig. 7B and C, respectively). TGFβ signaling promotes BRAFi/MEKi resistance and the activation of TFGβ is dependent on reduced regulation of SOX10 [30]. Real-time PCR analysis showed a dramatic downregulation in the expression level of the *SOX10* gene in both resistant melanoma cell lines versus control lines (Fig. 7E). Other protein involved in epithelial-mesenchymal transition is SOX2. It can modulate the level of MITF in melanoma cells [31]. We noticed a reduced level of this protein in examined resistant cells in comparison to control ones (Fig. 7D). Moreover, one of the EMT-activating transcription factors SLUG exhibits increased expression in both resistant melanoma cell lines (Fig. 7F). This elevation was about 600-fold higher in WM9 R and more than 50-fold higher in the Hs294T R cell line versus controls.

### Influence of resistance development on cytokine secretion by melanoma cells

**Fig. 8:**
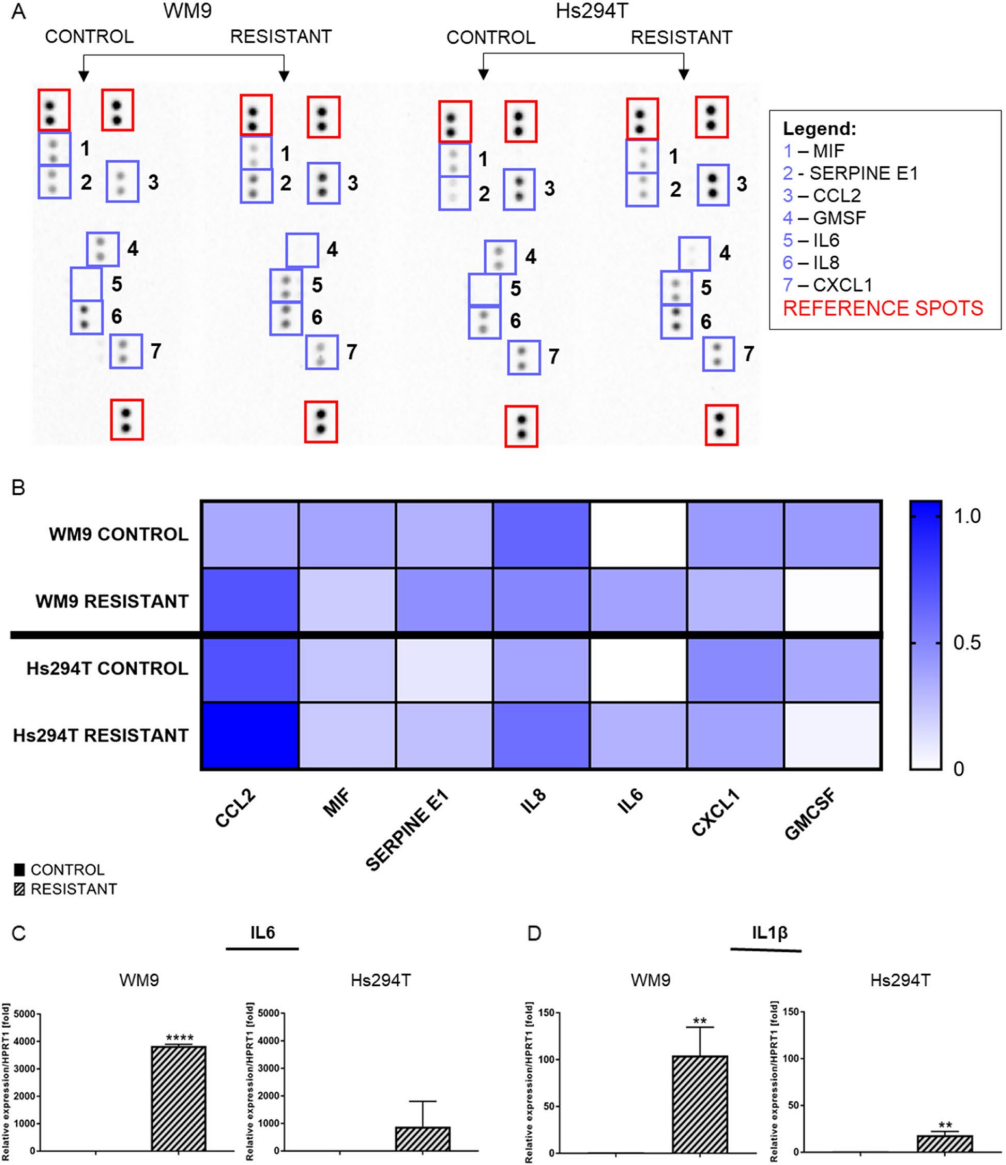
Cytokine secretion by control and resistant melanoma cell lines. Cell culture media collected from resistant and control cells were analyzed for cytokines via (**A**) signal (**B**) quantitative analysis. The results were normalized to reference spots and are shown in the form of a heatmap where darker blue indicates a higher signal intensity. Expression level of interleukins: (**C**) *IL6* and (**D**) *IL1β* in resistant and control melanoma WM9 and Hs294T cell lines. Real-time PCR was performed, and *HPRT1* constituted a reference gene. Control (CTRL) constitutes WM9 and Hs294T cells treated with regular media with DMSO at the concentration used for drug delivery. The graphs show average values from at least three independent experiments ± SD. Asterisks in the graphs indicate statistical significance (p) at the level of ** ≤ 0.01 and **** ≤ 0.0001. Abbreviations: MIF - macrophage migration inhibitory factor, CCL2 - C-C motif chemokine ligand 2, GMCSF - granulocyte/macrophage colony-stimulating factor, IL6 - interleukin 6; IL8 - interleukin 8, and CXCL1 - C-X-C motif chemokine ligand 1

The acquisition of drug resistance may be dependent on the composition of cell-secreted cytokines [24, 32]. A Proteome Profiler Human Cytokine Array Kit was used to verify whether, and to what extent they are secreted by control and resistant melanoma cells. The assay results (Fig. 8A) are presented in a form of a heatmap (Fig. 8B). From a panel of 36 cytokines, a subset was selected based on differences between the control and resistant melanoma cells. Increased levels of CCL2 (C-C motif chemokine ligand 2), serpine E1, and IL6 cytokines were observed in both resistant melanoma cell lines. Some cytokine levels were also reduced such as GMCSF (granulocyte/macrophage colony-stimulating factor), and CXCL1 (C-X-C motif chemokine ligand 1) in resistant cell lines in comparison to the controls. In the case of interleukin 8 (IL8) and MIF (macrophage migration inhibitory factor), divergent effects were observed; the level of IL8 was decreased in the case of WM9 R, while in Hs294T R we noted an increase. MIF level was reduced in WM9 R, while in the case of Hs294T R it did not changed.

Real-time PCR was performed to establish the mRNA level encoding *IL6* and *IL1β* in resistant and control melanoma cell lines (Fig. 8C and D, respectively). There was an increase in *IL6* expression level in both resistant cell lines; more than 3000-fold higher in WM9 R, and almost 1000-fold higher in the Hs294T R in comparison to their respective controls. Statistical significance was noted for the WM9 R cell line. This result is consistent with the data above. The expression of *IL1β* was increased in both resistant cell lines versus controls.

## Discussion

Combined therapy against BRAF and MEK is particularly effective in melanoma patients who have not previously been treated with BRAF inhibitors [33]. Unfortunately, drug resistance still develops relatively quickly when a combination of both inhibitors is used. In this study melanoma cell lines resistant to treatment with BRAF and MEK inhibitors were obtained and characterized. To date, the effect of BRAF inhibitors on melanoma cells is quite well described. We have also contributed to the field with research based on melanoma cells treated with vemurafenib [15]. However, it is important to note, that melanoma cells differ in terms of the features that appear in connection with the acquisition of resistance to BRAFi monotherapy and a mixture of BRAF/MEK inhibitors. Firstly, WM9 cells resistant to the drug combination showed increased levels of ALCAM protein, while this effect was not observed in cells resistant only to the BRAF inhibitor [15]. Moreover, melanoma cells resistant to cobimetinib and vemurafenib showed a statistically significant increase in the number of cells in the G0/G1 phase of the cell cycle and a decrease in the number of cells in the S phase of the cell cycle; cells resistant to vemurafenib alone did not reveal significant differences in the number of cells in both mentioned phases in comparison to the control ones (data not shown). Additionally the level of ErbB2 expression was increased in cells resistant to the combination of inhibitors (in relation to the control), while in cells resistant to vemurafenib it actually decreases (data not shown). Other researchers also detected significant changes like much more pronounced decrease in proliferation and activation of pERK, in the melanoma cells treated with BRAFi/MEKi drug combination in comparison to BRAFi-based monotherapy [34].

The development of resistant cell lines was a big challenge because it is a time-consuming process and not all melanoma cell lines tolerate treatment with combined inhibitors for a long period of time. We used the inhibitors in equal concentrations. In clinical settings, the combination of vemurafenib and cobimetinib is used in a ratio of 20:1, which is associated with numerous side effects associated with the use of the mentioned MEK kinase inhibitor. Our studies aimed to understand the molecular mechanisms responsible for resistance to both drugs – BRAF and MEK inhibitors in melanoma models in vitro. They cannot be directly translated into clinical approach. Melanoma cells resistant to BRAFi/MEKi showed significantly higher viability and phosphorylation of ERK1/2 even at high concentrations of the combined inhibitors versus control. We and others also noticed recovery of ERK1/2 phosphorylation after the use of a BRAF inhibitor in resistant melanoma cell lines [15, 35].

It is well known that reactivation of pathways such as MAPK or PI3K/AKT may be one of the causes underlying the emergence of drug resistance. They are also involved in angiogenesis, stem-like phenotype, and an EMT [36, 37]. We observed an increased pAKT/AKT ratio in both resistant melanoma lines. In melanoma, AKT mutations can lead to increased induction of this pathway [38]. Some reports indicate the phosphorylation of the ERK protein by the AKT pathway [35]. We also noted elevated activation of two components of the MAPK pathway: p38 and JNK in both cell lines. In most cancers, elevated level of p38 inhibits ERK activation through negative feedback. A different situation occurs in melanoma where both ERK1/2 and p38 may be activated simultaneously through a positive feedback loop [39]. Furthermore, an early increase in p38 after BRAFi therapy was also demonstrated, which may indicate that p38 is a mediator of the adaptive response of melanoma cells to the treatment [40]. The JNK pathway has been also shown to be involved in drug resistance occurring in several types of cancers including melanoma [41]. Increased levels of JNK were observed in some melanoma cell lines, and simultaneous inhibition of the RAF and JNK pathways resulted in synergistic induction of apoptosis in cancer cells [42].

Finally, we noted an increase in *NRAS* expression in both resistant melanoma cell lines. Some reports indicate that overexpression of this gene could be connected with enhanced activation of all three aforementioned pathways in melanoma cell lines resistant to vemurafenib treatment. Moreover, it was shown that NRAS suppression resulted in increased sensitivity to vemurafenib and reduced activation of the p38 and JNK pathways in vemurafenib-resistant melanoma cell lines [43]. We detected also increased level of MITF in examined resistant cells. MITF is a melanocyte lineage-specific transcription factor that is required for melanoblast survival, plays an important role in melanocyte development, and regulates the expression of pigment-producing enzymes [44]. Some studies reported that a MITF^high^ state is associated with MAPKi therapy resistance and poor prognosis [45–47], whereas others show that a MITF^low^ state in combination with high expression levels of several RTKs (e.g. AXL) is responsible for therapy resistance [48]. MITF^high^ tumors were shown to be responsive to MAPKi, however, tumors that were initially MITF^low^ upregulate MITF upon treatment, leading to the development of resistance [49]. Smith et al. indicated also that MITF is required to produce resistance to MEK inhibitor–induced cell death and its increased expression allows melanoma cells to escape the proapoptotic effects of MEK inhibition [45].

The significant role of receptor tyrosine kinases in the acquisition of resistance by melanoma cells to BRAFi or BRAFi/MEKi treatment was demonstrated earlier. Importantly, proteolytic shedding of cell surface receptors may occur as a part of the negative feedback loop, and this may limit intracellular signaling of RTKs. However, this process was shown to be inhibited in the presence of BRAF inhibitor [38]. The qPCR analysis revealed increased expression of *EGFR* (HER1) and *HER2* in both resistant melanoma cell lines in comparison to the control ones. EGFR activity is associated with cell drug resistance [50–53]. Its overexpression was observed in 6 of 16 biopsies derived from patients with BRAFi/MEKi-resistant melanomas and may be associated with elevated invasiveness of melanoma cells [54]. Increased expression of *EGFR* accompanied by reduced expression of *HER3* was characteristic of the invasive subtype of BRAFi-resistant melanoma cells [55]. This could explain the extremely low expression of this second receptor in the obtained resistant lines.

In addition, and more important in the context of this study, suppression of SOX10 leads to activation of TGFβ signaling and consequent upregulation of EGFR and PDGFRβ, that was associated with BRAFi/MEKi treatment [54]. Increased levels of PDGFRβ have often been described in cases when drug resistance to BRAFi occurred [35, 56, 57]. All of the effects described above were also observed in the obtained resistant melanoma cell lines. The last receptor whose expression is increased in resistant melanoma cells is MET. An increased level of MET in cells resistant to combined therapy was observed by another research team [58], while elevated expression of *EGFR* and *MET* was noticed by us previously in vemurafenib-resistant A375 and WM9 cells [15]. Elevated levels of the above-mentioned RTKs can activate ERK and AKT and consequently lead to early disease progression [59].

The project further investigated ATP-binding cassette (ABC) transporters, which are frequently expressed in cancer cells and fulfill key functions in the distribution, absorption, excretion, and gaining resistance to the drugs, including vemurafenib [60]. Generally, to date, only a few members of the ABC transporters family have been described in melanoma, and their role remains unknown and requires further investigation. ABCA1 is mainly described as a transporter responsible for cholesterol efflux. However, ABCA1 seems to be somehow involved in the progression of melanoma, because its increased expression was demonstrated in more advanced tumors; it was also correlated with a shorter overall survival. Moreover, the loss of activity of the ABCA1 transporter resulted in a lower potential for migration in Hs294T cells [61]. Another transporter most often described in the context of multidrug resistance in cancer cells is ABCG2. Its role in transporting drugs into extracellular fluids [62] as well as in conferring resistance to various anti-cancer drugs has been described [63, 64]. This transporter has been reported as the likely most common in melanoma [65]. Vemurafenib is one of the substrates of ABCG2 [66], and it was suggested that this transporter could influence BRAF resistance acquisition in melanoma cells [60]. Interestingly, it has been reported that ABCG2 activity can be regulated by the PI3K/AKT pathways [67]. In the case of ABCG2, to the best of our knowledge, this is the first report of an increased level of this transporter in cells resistant to BRAFi/MEKi. The last examined transporter, ABCC2, is responsible for drugs’ efflux and is described in the literature in the context of the emergence of drug resistance [66] and in the occurrence in melanoma [68]. However, to the best of our knowledge, this is the first study to demonstrate its association with BRAFi/MEKi resistance development.

Another family of tested proteins was human cytochrome P450 enzymes (CYPs). They play key roles in the metabolism of various exogenous and endogenous substrates. We detected an increased level of CYP1A1, which has been shown to activate/inactivate anti-cancer agents [69]. The role of CYP1A1 in metabolizing an EGFR inhibitor gefitinib [70] and a BRAF inhibitor vemurafenib [71] has also been shown. However, the effect of BRAFi/MEKi therapy on the expression of this cytochrome has not been previously investigated.

We observed that resistant melanoma cells proliferate much slower than control ones. Decreased melanoma cell proliferation has also been shown by others when a MEK inhibitor was used as a monotherapy and with combined BRAFi/MEKi therapy [72]. Moreover, analysis of cell cycle phases distribution revealed an increase in the G1/G0 and S phase in both resistant lines, which could partially explain the reduced proliferation rate of obtained resistant cells. We also noticed changes in the level of proteins involved in cell cycle regulation. CDK6 plays a pivotal role in the progression of the cell cycle, and we observed that resistant cells have decreased level of this protein, that may be connected with reduced proliferation rate as well as cell cycle arrest in the G1 phase [73]. The p18, p21, and p27 proteins are inhibitors of cell cycle progression [74, 75]. Their levels were reduced in our resistant cells, which may be surprising. It was also indicated that reduced level of p21 and p27 may be associated with increased tumorigenesis and reduced lifespan in mice [76], suffering from cancer, including melanoma [77, 78]. p21 is known to attenuate breast tumor EMT and CSC-like gene expression, and its low expression is related to reduced sensitivity of melanoma cells towards targeted therapies [79, 80]. Furthermore, it was shown that p27 is downregulated in lung cancer cells resistant to MET inhibitor [81]. Moreover, it was demonstrated that p-Akt is elevated in examined resistant cells, where it phosphorylates and inhibits p21 and p27 activity [82].

CSCs can support tumor recurrence and progression [83]. The administration of anti-cancer drugs may lead to the induction of EMT and pathways responsible for self-renewal in CSCs, as well as an increase in expression of drug transporters or detoxification proteins in these cells [28]. We observed upregulated expression of *CD24* and *NANOG*, as well as elevated level of ALCAM protein in both resistant lines. All of the aforementioned proteins are CSC markers. Increased *CD24* expression was shown to be associated with decreased sensitivity to BRAFi in resistant melanoma cells [84], while elevated ALCAM levels were observed in the vemurafenib-resistant melanoma cell line [15]. NANOG has been described in the context of CSCs and drug resistance in various cancers, and it has also been shown that its overexpression was associated with an increased ability to form spheroids in vitro [85]. The resistant melanoma also exhibited that ability. The development of spheroids during cell growth is a characteristic feature of CSCs [26–28].

After acquiring resistance to BRAFi/MEKi treatment, cells presented an elongated morphology and spindle-like-shape. Such morphological changes are features related to the EMT process [26, 29], which — besides its role during carcinoma progression [26] — is also a factor contributing to drug resistance [26, 28, 83]. Our observations are consistent with other reports indicating that melanoma cells treated with vemurafenib and trametinib (another MEK inhibitor) exhibit morphological changes [86]. The EMT process can be induced by transcription factors such as *SLUG*, as well as chemokines and pro-inflammatory cytokines like IL6 or IL1β [26]. Increased expression of *SLUG* and interleukins *6* and *1β* were observed in both resistant melanoma cell lines. On the other hand, TGFβ receptors I and III were elevated at the protein level with a concomitant decrease in *SOX10* expression in derived resistant lines. These results are consistent with reports indicating that reduced level of SOX10 influences the activation of TGFβ and further leads to EMT induction in BRAFi/MEKi-resistant cells [30]. Loss of *SOX10* results in a reduced proliferation rate [87]. A positive correlation between the level of SOX2 and the ability of melanoma cells to invade and acquire resistance to treatment is well known [88, 89]. However, we detected reduced expression of this protein in resistant cells. It was shown that in lung cancer SOX2 down-regulation promotes mesenchymal phenotype and mediates resistance of tumor cells to anti-cancer drugs. This phenomenon was related to the interaction between SOX2 and TGF-β, which reduced the expression level of SOX2 to induce EMT and promote metastasis of lung cancer cells [90, 91].

Because of the influence of cytokines on the EMT process, we have conducted a screening assay estimating their level in a conditioned medium derived from tested cell lines. An increase in the secretion of several cytokines has been demonstrated including CCL2 and serpine E1. In contrast, the level of secretion of some of them was decreased (GMCSF, and CXCL1) or differed between obtained resistant lines (MIF, and IL8). In addition, qPCR revealed increased expression of *IL6* and *IL1β*. Altered cytokine expression has been also described as a factor contributing to the mechanism of drug resistance [59]. IL6 has been identified as a driver of drug resistance [32], while increased level of CCL2 was detected in resistant melanoma cells after treatment with a BRAF inhibitor [92]. The *SERPINE E1* gene encodes the plasminogen activator inhibitor 1 (PAI-1) protein, and its secretion may influence the chemoresistance of melanoma cells [93]. Finally, increased production of proinflammatory cytokines and chemokines by cancer cells was shown to be induced by NRAS oncoprotein [26], which elevated expression was also observed in resistant cell lines. On the other hand, resistant cells secrete reduced amount of GMCSF, which recruits dendritic cells and in turn present tumor antigens to cytotoxic T lymphocytes, thus inducing a systemic tumor-directed immune response [94].

## Conclusions

Obtained resistant melanoma cells exhibit increased activation of signaling pathways, including JNK, which raised activation in resistant to BRAFi/MEKi melanoma cells is demonstrated here for the first time. Surprisingly, we also observed an increased level of MITF in the tested resistant cells. Moreover, expression of some RTK family receptors is raised in these cells, while the level of HER3 was reduced, what, together with elevated EGFR level, is characteristic of the invasive subtype of BRAFi-resistant melanoma cells. ABC transporters and CYP1A1 protein level was also upregulated in resistant cells. Moreover, the elevated levels of ABCA1, ABCC2 and ABCG2 were here shown for the first time in BRAFi/MEKi resistant cells. Both resistant cell lines show also the characteristics of cancer stem cells and display features related to epithelial-mesenchymal transition (EMT). The EMT process is closely related to the CSC state and both support the emergence of drug resistance, similar to changed cytokine secretion. These features of resistant cells may contribute to their increased ability to survive and form metastases. They could also constitute the basis for selecting new and potentially therapeutic targets.

### Supplementary Information

Supplementary Material 1.

Supplementary Material 2.

## Data Availability

The authors declare that the data supporting the findings of this study are available within the paper. Any raw data files are available from the corresponding author upon reasonable request.
