# Supplementary figures and images for: Characterization of two melanoma cell lines resistant to BRAF/MEK inhibitors (vemurafenib and cobimetinib)
Source: Cell Commun Signal. 2024 Aug 23;22:410. doi: 10.1186/s12964-024-01788-3 (PMC11342534; doi:10.1186/s12964-024-01788-3)

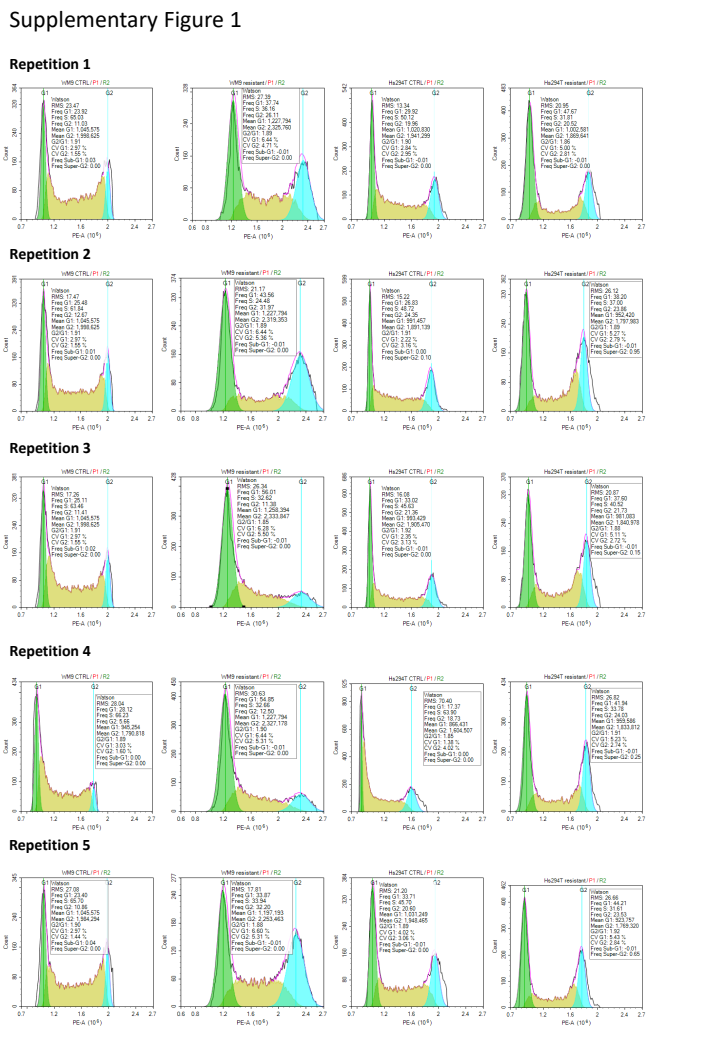

Supplement: Supplementary file 1 — Supplementary Material 1. [file 12964_2024_1788_MOESM1_ESM.tif]
