# Supplementary material for: Characterization of two melanoma cell lines resistant to BRAF/MEK inhibitors (vemurafenib and cobimetinib)
Source: Cell Commun Signal. 2024 Aug 23;22:410. doi: 10.1186/s12964-024-01788-3 (PMC11342534; doi:10.1186/s12964-024-01788-3)

Western blotting membranes – raw images

pERK WM9 (Fig.1.)

Repetition 1

pERK

WM9 CTRL

CTRL DMSO, 0.005C+0,005V, 0.01+0.01V, 0.4C+0.4V, 0.8C+0.8V, 2C+2V

WM9 R

CTRL DMSO, 0.005C+0,005V, 0.01+0.01V, 0.4C+0.4V, 0.8C+0.8V, 2C+2V

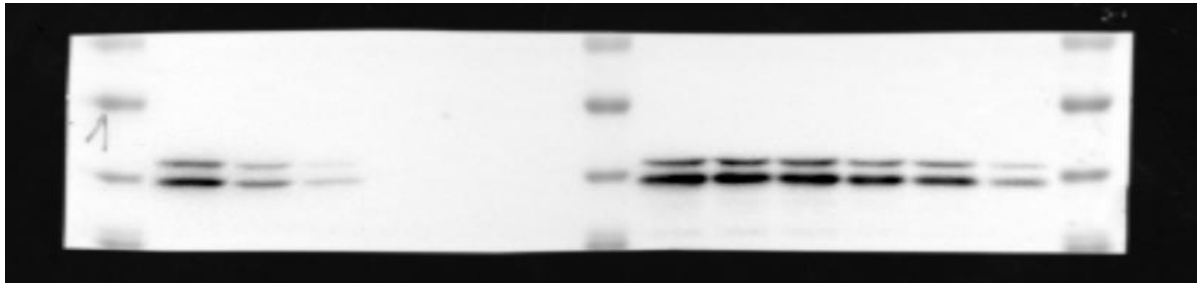

Ponceau Staining

WM9 CTRL

CTRL DMSO, 0.005C+0,005V, 0.01+0.01V, 0.4C+0.4V, 0.8C+0.8V, 2C+2V

WM9 R

CTRL DMSO, 0.005C+0,005V, 0.01+0.01V, 0.4C+0.4V, 0.8C+0.8V, 2C+2V

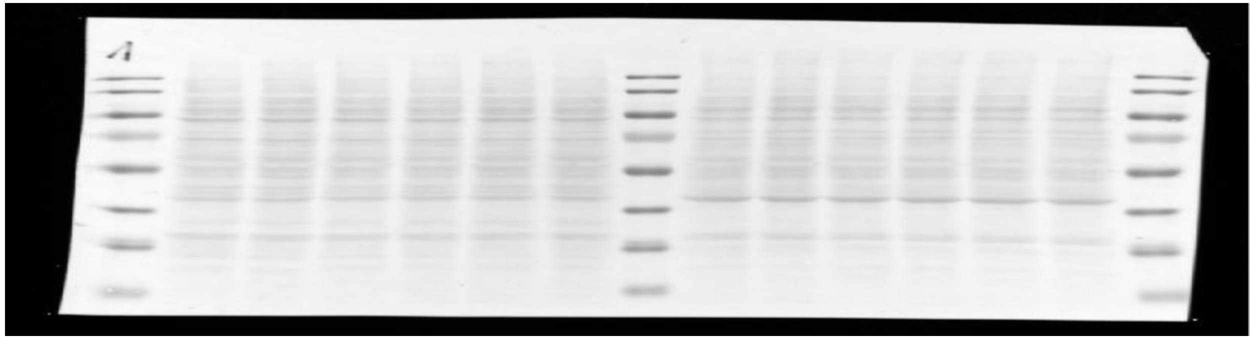

Repetition 2

pERK

WM9 CTRL

CTRL DMSO, 0.005C+0,005V, 0.01+0.01V, 0.4C+0.4V, 0.8C+0.8V, 2C+2V

WM9 R

CTRL DMSO, 0.005C+0,005V, 0.01+0.01V, 0.4C+0.4V, 0.8C+0.8V, 2C+2V

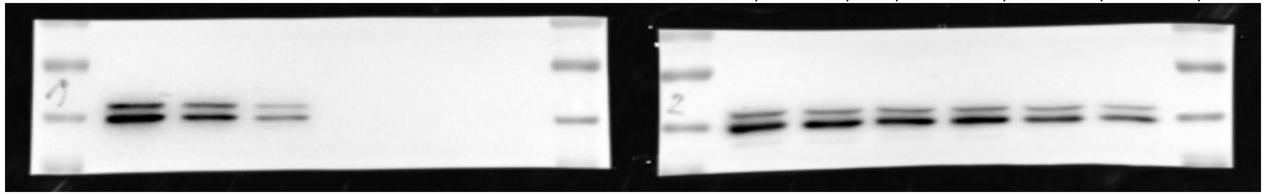

Ponceau Staining

WM9 CTRL

CTRL DMSO, 0.005C+0,005V, 0.01+0.01V, 0.4C+0.4V, 0.8C+0.8V, 2C+2V

WM9 R

CTRL DMSO, 0.005C+0,005V, 0.01+0.01V, 0.4C+0.4V, 0.8C+0.8V, 2C+2V

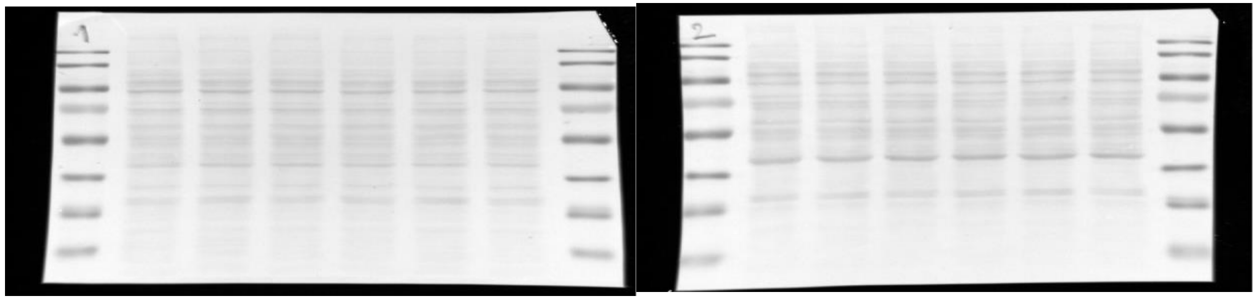

Repetition 3

pERK

WM9 CTRL

CTRL DMSO, 0.005C+0,005V, 0.01+0.01V, 0.4C+0.4V, 0.8C+0.8V, 2C+2V

WM9 R

CTRL DMSO, 0.005C+0,005V, 0.01+0.01V, 0.4C+0.4V, 0.8C+0.8V, 2C+2V

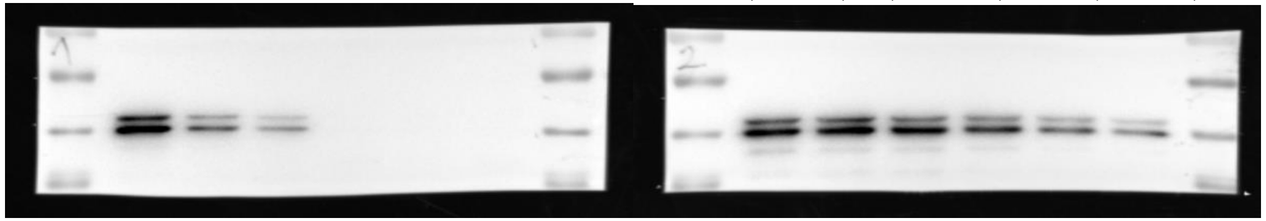

Ponceau Staining

WM9 CTRL

CTRL DMSO, 0.005C+0,005V, 0.01+0.01V, 0.4C+0.4V, 0.8C+0.8V, 2C+2V

WM9 R

CTRL DMSO, 0.005C+0,005V, 0.01+0.01V, 0.4C+0.4V, 0.8C+0.8V, 2C+2V

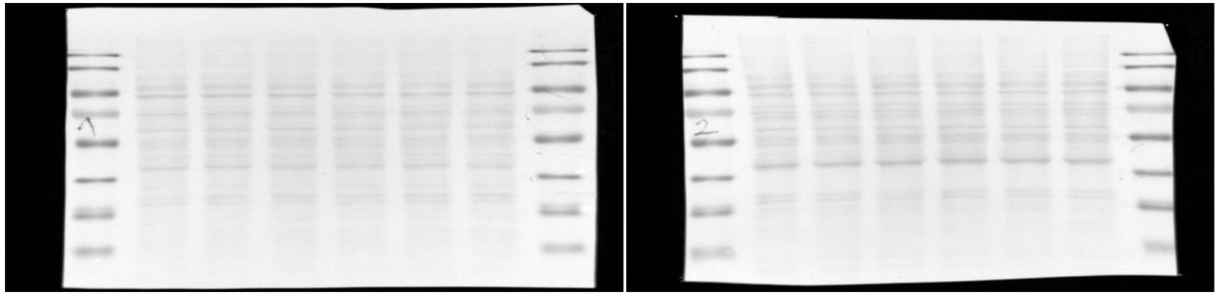

pERK Hs294t (Fig.1.)

Repetition 1

pERK

Hs294T CTRL

CTRL DMSO, 0.005C+0,005V, 0.01+0.01V, 0.4C+0.4V, 0.8C+0.8V, 2C+2V

Hs294T R

CTRL DMSO, 0.005C+0,005V, 0.01+0.01V, 0.4C+0.4V, 0.8C+0.8V, 2C+2V

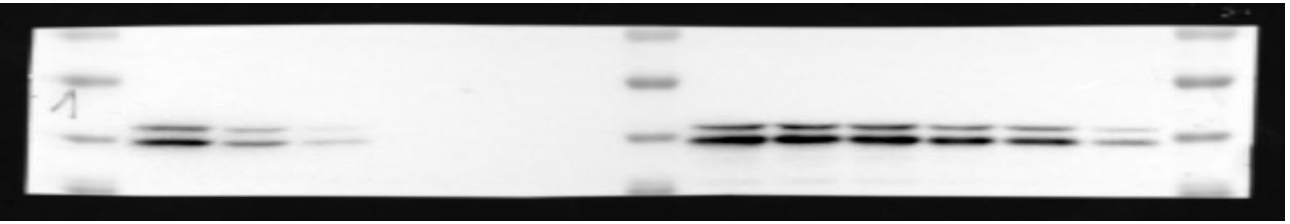

Ponceau Staining

Hs294T CTRL

CTRL DMSO, 0.005C+0,005V, 0.01+0.01V, 0.4C+0.4V, 0.8C+0.8V, 2C+2V

Hs294T R

CTRL DMSO, 0.005C+0,005V, 0.01+0.01V, 0.4C+0.4V, 0.8C+0.8V, 2C+2V

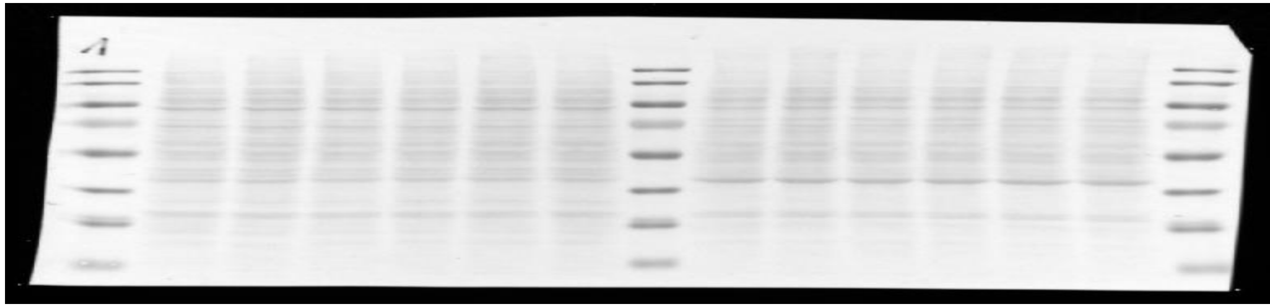

Repetition 2

pERK

Hs294T CTRL

CTRL DMSO, 0.005C+0,005V, 0.01+0.01V, 0.4C+0.4V, 0.8C+0.8V, 2C+2V

Hs294T R

CTRL DMSO, 0.005C+0,005V, 0.01+0.01V, 0.4C+0.4V, 0.8C+0.8V, 2C+2V

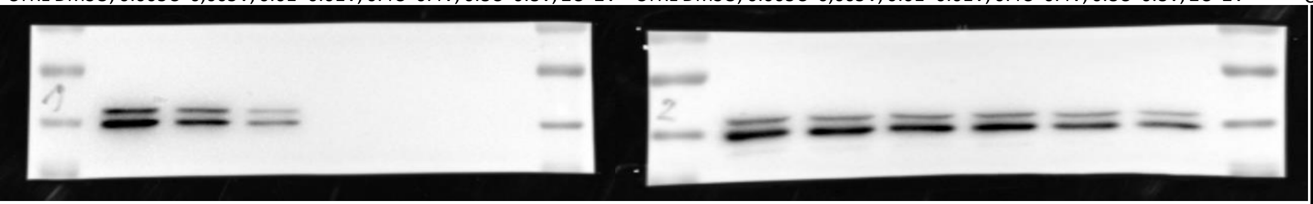

Ponceau Staining

Hs294T CTRL

CTRL DMSO, 0.005C+0,005V, 0.01+0.01V, 0.4C+0.4V, 0.8C+0.8V, 2C+2V

Hs294T R

CTRL DMSO, 0.005C+0,005V, 0.01+0.01V, 0.4C+0.4V, 0.8C+0.8V, 2C+2V

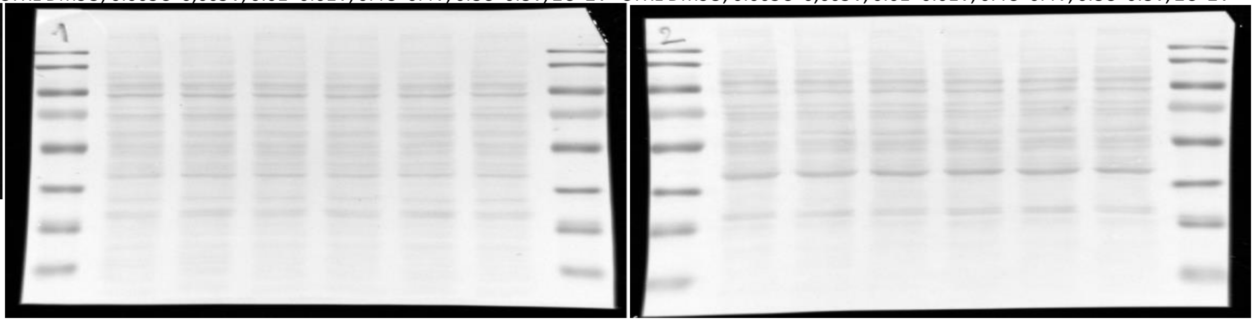

Repetition 3

pERK

Hs294T CTRL

CTRL DMSO, 0.005C+0,005V, 0.01+0.01V, 0.4C+0.4V, 0.8C+0.8V, 2C+2V

Hs294T R

CTRL DMSO, 0.005C+0,005V, 0.01+0.01V, 0.4C+0.4V, 0.8C+0.8V, 2C+2V

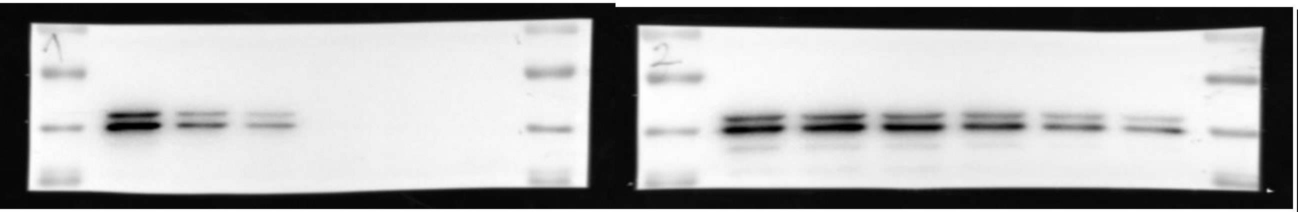

Ponceau Staining

Hs294T CTRL

CTRL DMSO, 0.005C+0,005V, 0.01+0.01V, 0.4C+0.4V, 0.8C+0.8V, 2C+2V

Hs294T R

CTRL DMSO, 0.005C+0,005V, 0.01+0.01V, 0.4C+0.4V, 0.8C+0.8V, 2C+2V

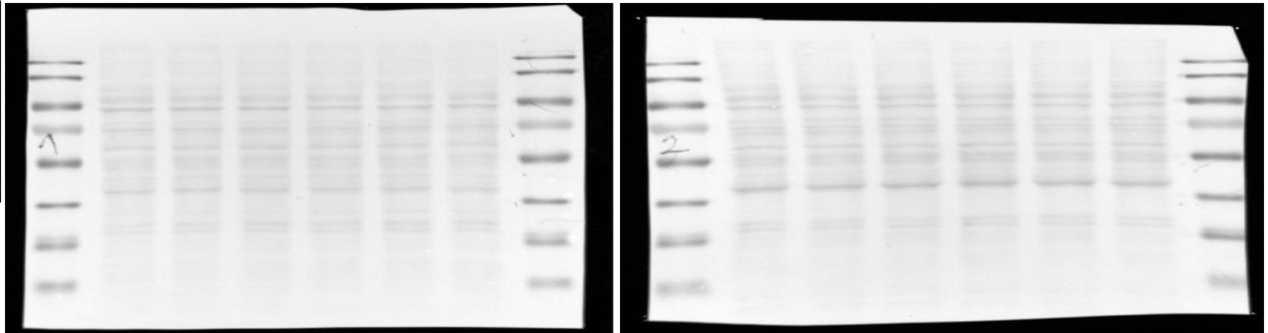

AKT/pAKT WM9 (Fig.2.) CTRL- control R- resistant

AKT **Repetition 1**

CTRL R

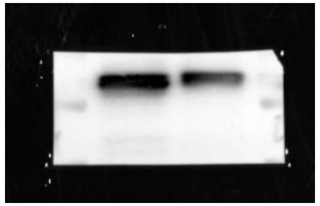

Ponceau Staining

CTRL R

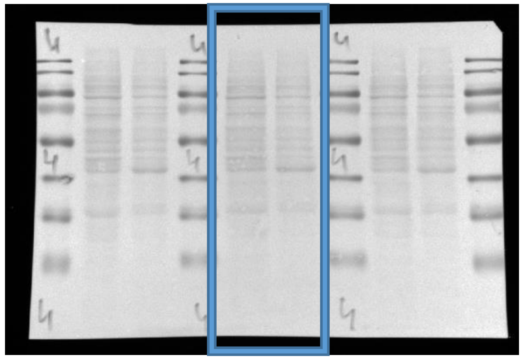

AKT **Repetition 2 and 3**

CTRL R CTRL R

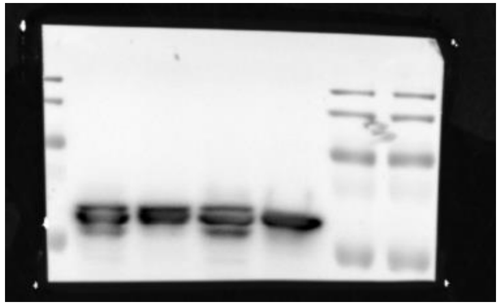

Ponceau Staining

CTRL R CTRL R

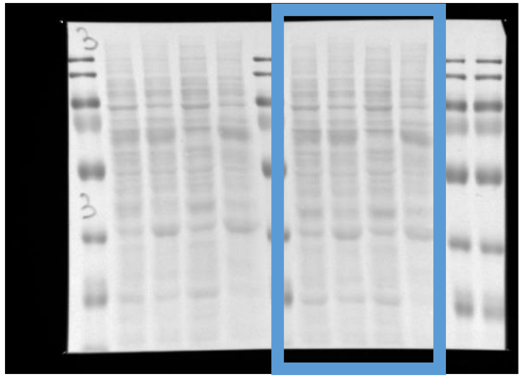

pAKT **Repetition 1**

CTRL R

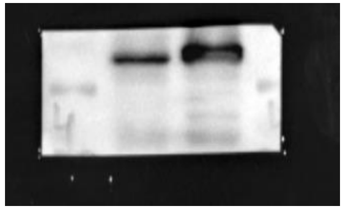

Ponceau Staining

CTRL R

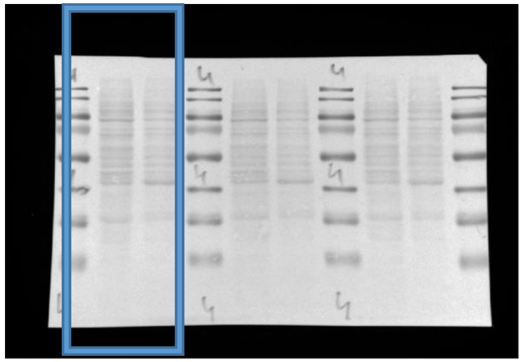

pAKT **Repetition 2 and 3**

CTRL R CTRL R

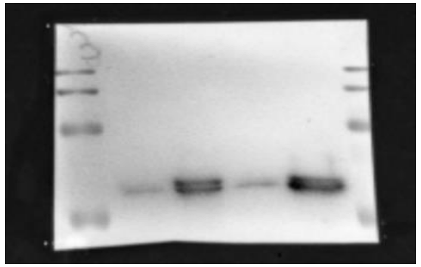

Ponceau Staining

CTRL R CTRL R

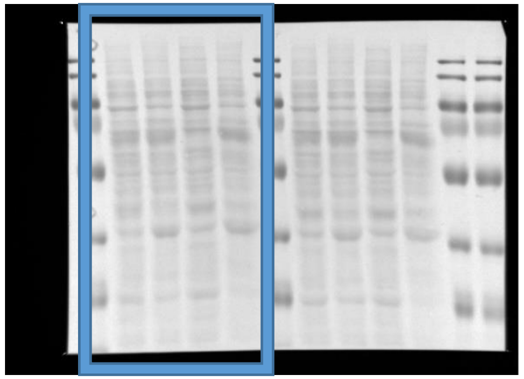

AKT/pAKT Hs294T (Fig.2.) CTRL- control R- resistant

AKT **Repetition 1,2,3** **Ponceau Staining**

CTRL R CTRL R CTRL R

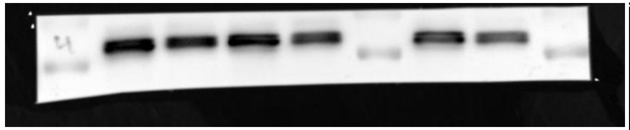

CTRL R CTRL R CTRL R

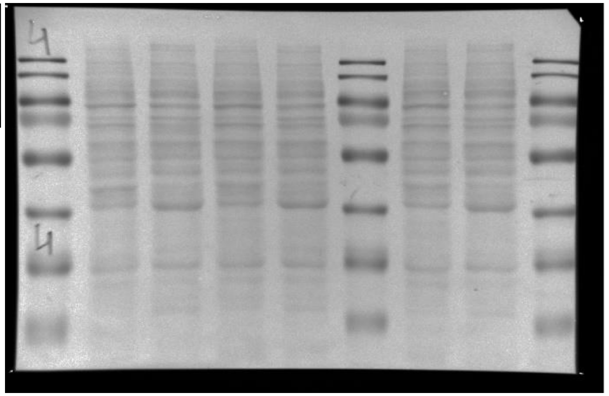

pAKT **Repetition 1,2,3** **Ponceau Staining**

CTRL R CTRL R CTRL R

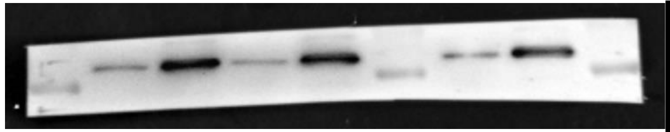

CTRL R CTRL R CTRL R

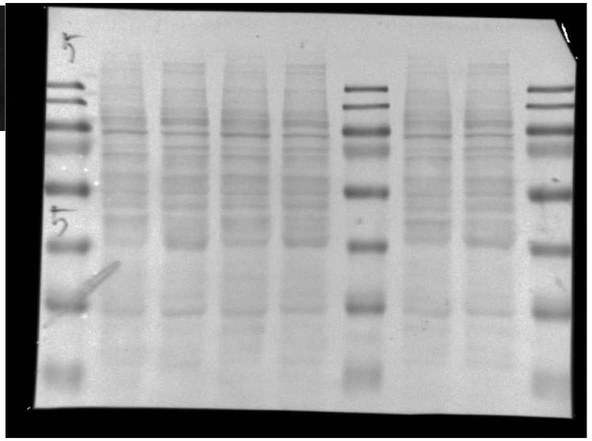

JNK/pJNK WM9 (Fig.2.) CTRL- control R- resistant

JNK **Repetition 1,2,3**

CTRL R CTRL R CTRL R

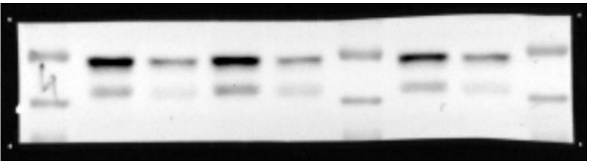

Ponceau Staining

CTRL R CTRL R CTRL R

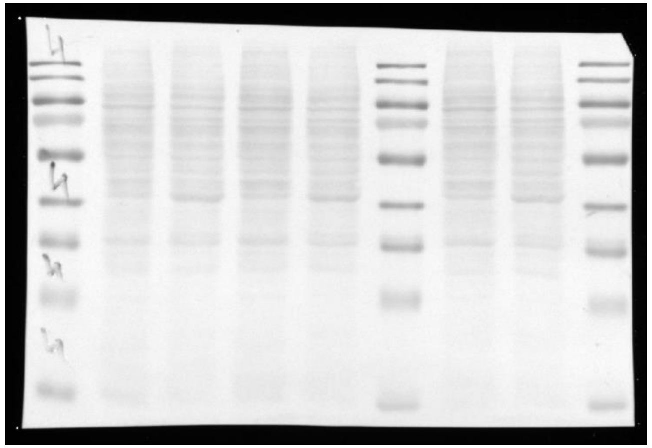

pJNK **Repetition 1,2,3**

CTRL R CTRL R CTRL R

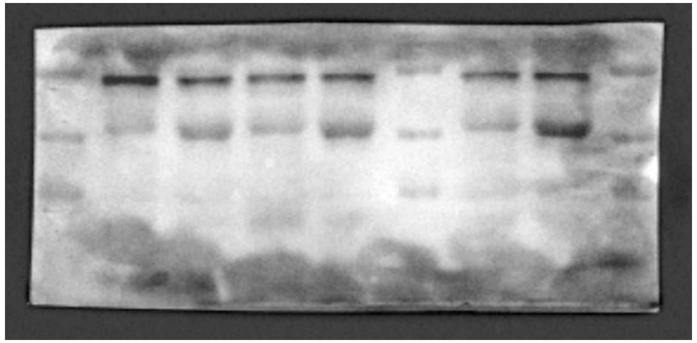

Ponceau Staining

CTRL R CTRL R CTRL R

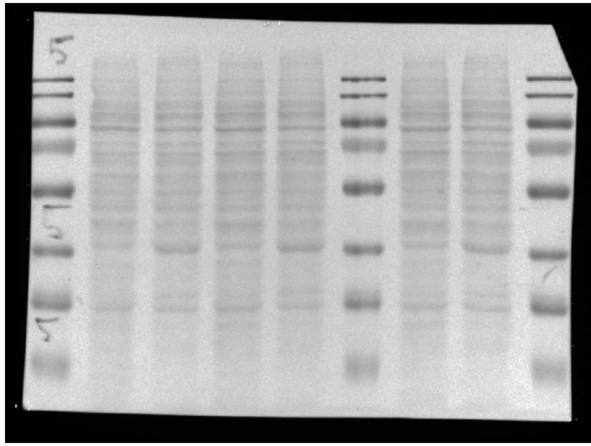

JNK/pJNK Hs294T (Fig.2.) CTRL- control R- resistant

JNK **Repetition 1,2,3** **Ponceau Staining**

CTRL R CTRL R CTRL R

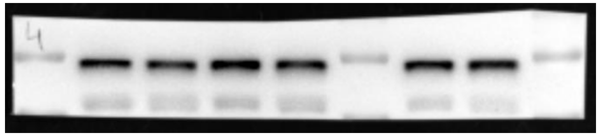

CTRL R CTRL R CTRL R

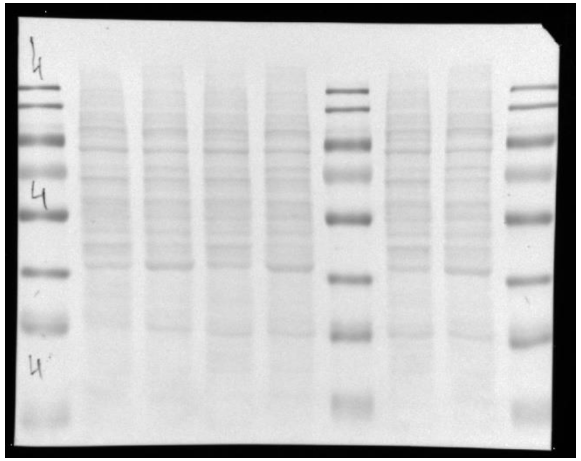

pJNK **Repetition 1,2,3** **Ponceau Staining**

CTRL R CTRL R CTRL R

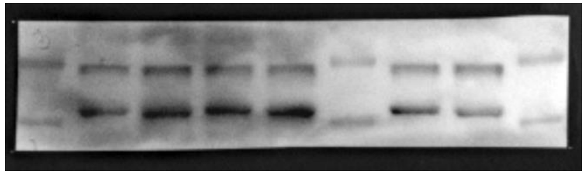

CTRL R CTRL R CTRL R

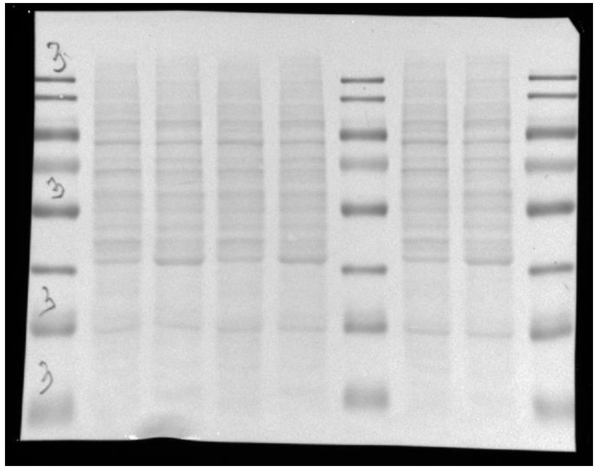

p38/ WM9 (Fig.2.)

p38

CTRL R

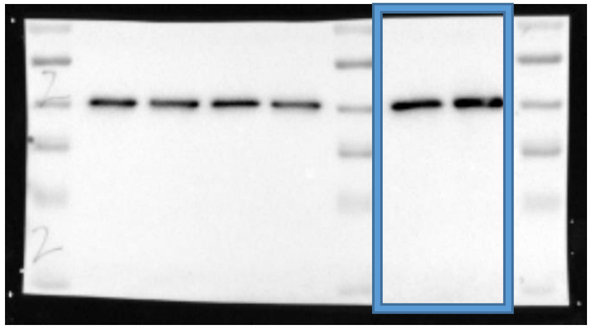

Repetition 1

Ponceau Staining

CTRL R

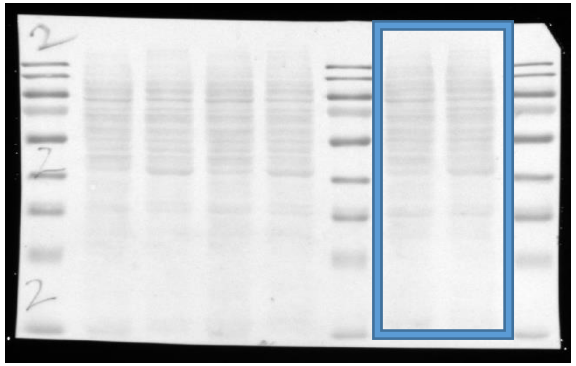

p38

CTRL R

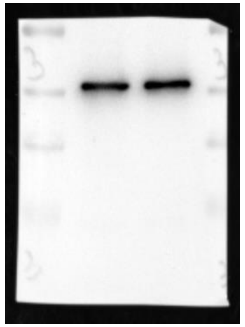

Repetition 2

Ponceau Staining

CTRL R

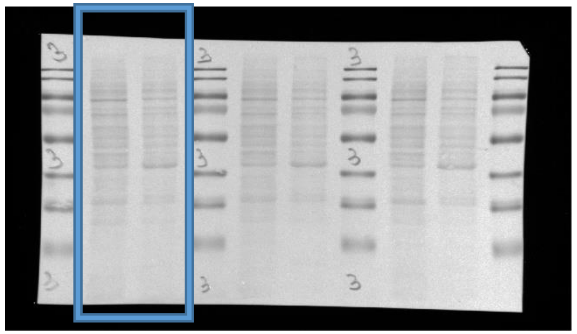

p38

CTRL R

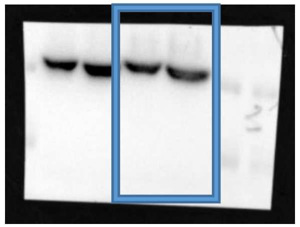

Repetition 3

Ponceau Staining

CTRL R

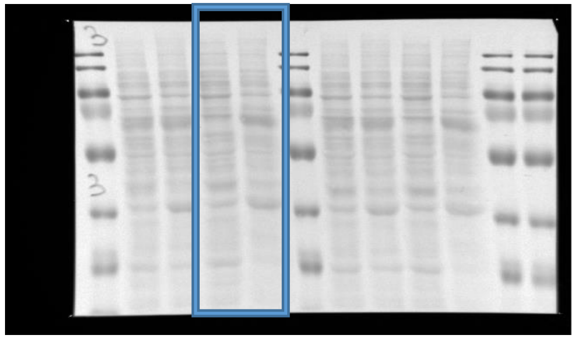

pp38/ WM9 (Fig.2.)

pp38

CTRL R

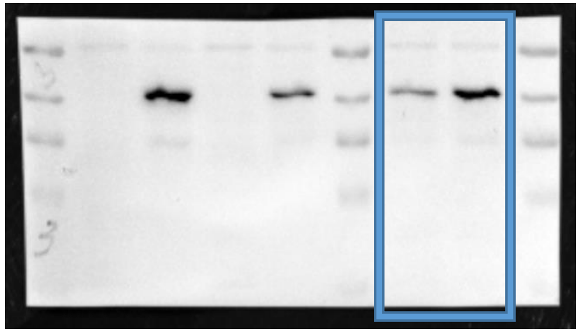

Repetition 1

Ponceau Staining

CTRL R

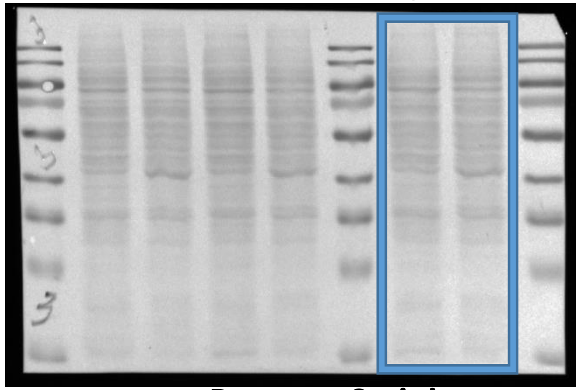

pp38

CTRL R

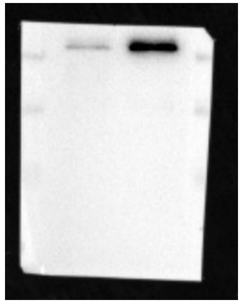

Repetition 2

Ponceau Staining

WM9 CTRL WM9 R

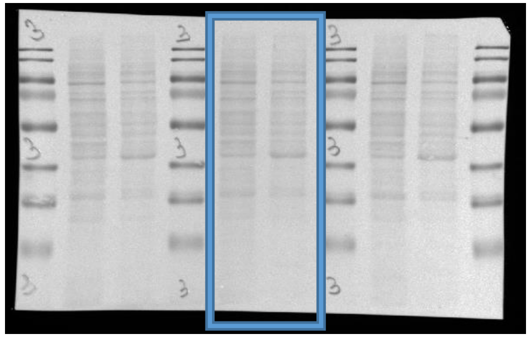

pp38

CTRL R

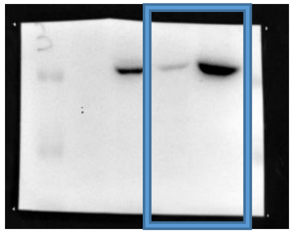

Repetition 3

Ponceau Staining

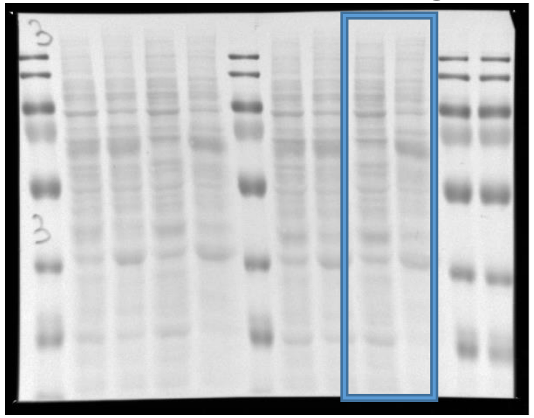

pp38/p38 Hs294T (Fig.2.)   CTRL- control   R- resistant

p38   **Repetition 1,2,3**   **Ponceau Staining**

CTRL R CTRL R CTRL R   CTRL R CTRL R CTRL R

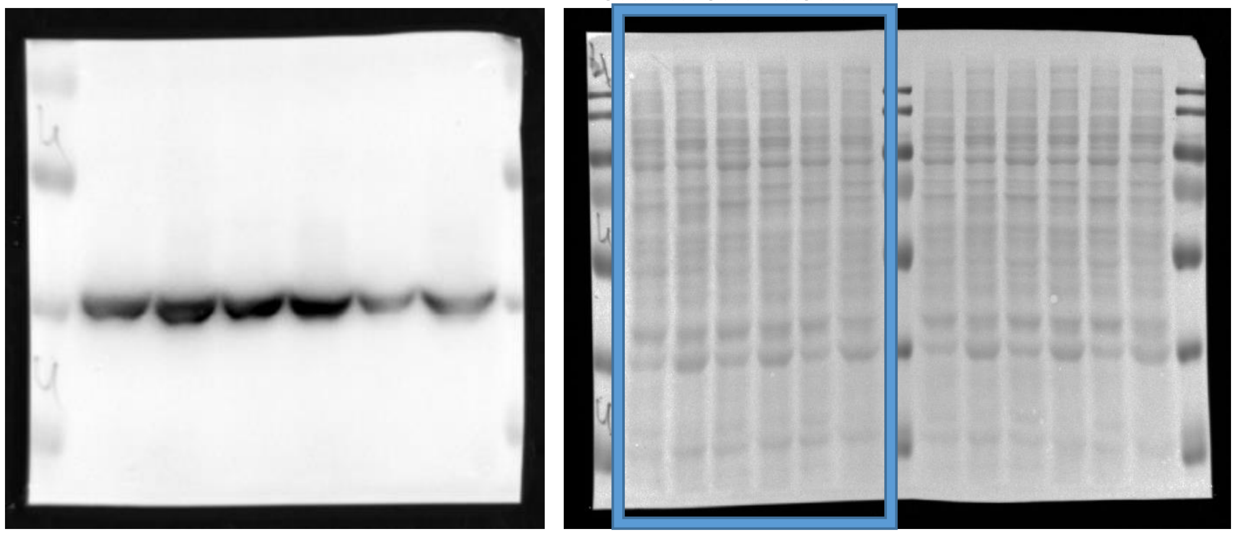

pp38   **Repetition 1,2,3**   **Ponceau Staining**

CTRL R CTRL R CTRL R   CTRL R CTRL R CTRL R

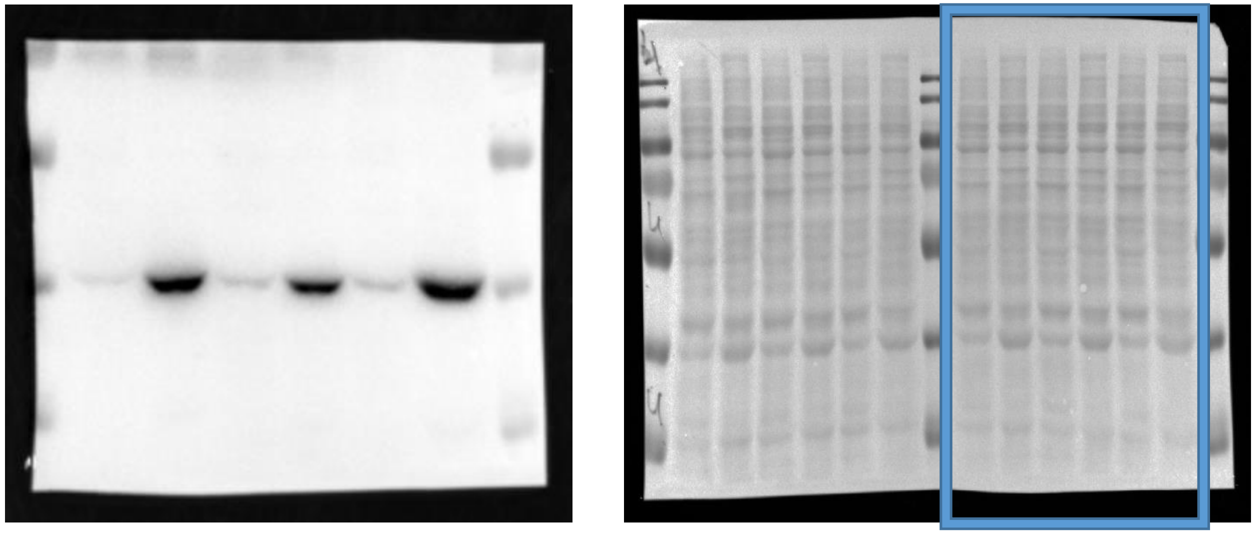

CYP1A1 WM9 (Fig.4.) CTRL- control R- resistant

CYP1A1 **Repetition 1,2**

CTRL R CTRL R

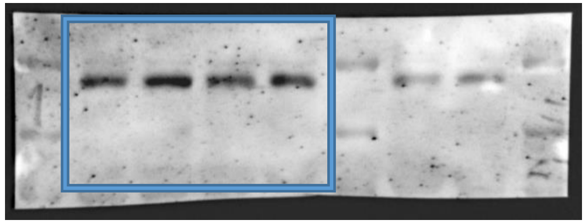

Ponceau Staining

CTRL R CTRL R

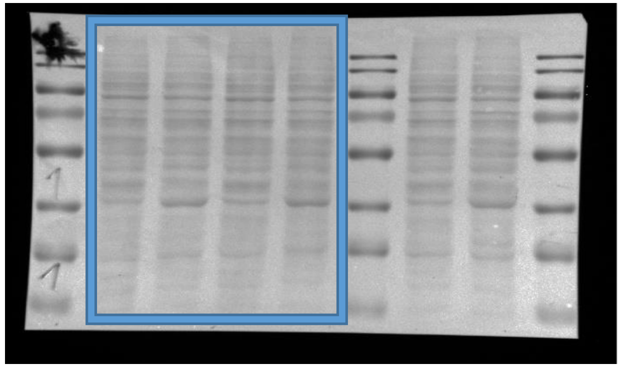

CYP1A1 **Repetition 3**

CTRL R

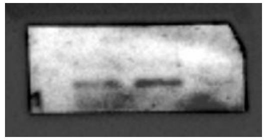

Ponceau Staining

CTRL R

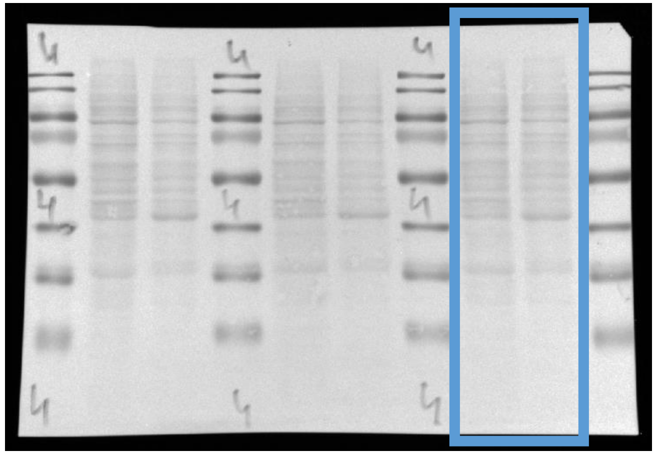

CYP1A1 Hs294T (Fig.4.) CTRL- control R- resistant

CYP1A1 **Repetition 1,2,3** **Ponceau Staining**

CTRL R CTRL R marker CTRL R

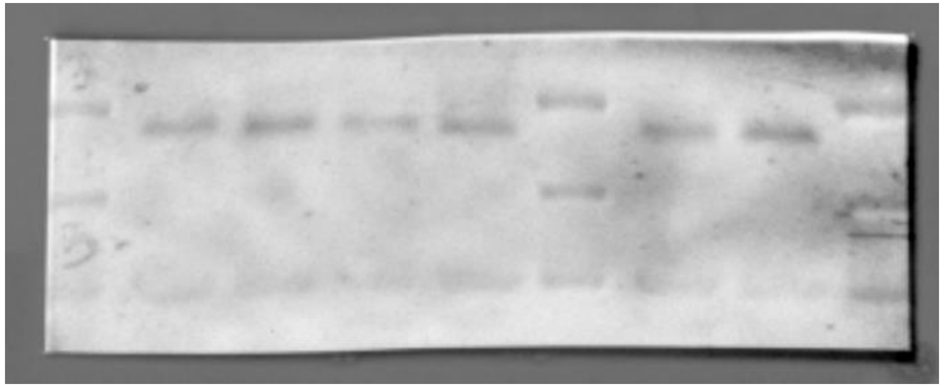

CTRL R CTRL R CTRL R

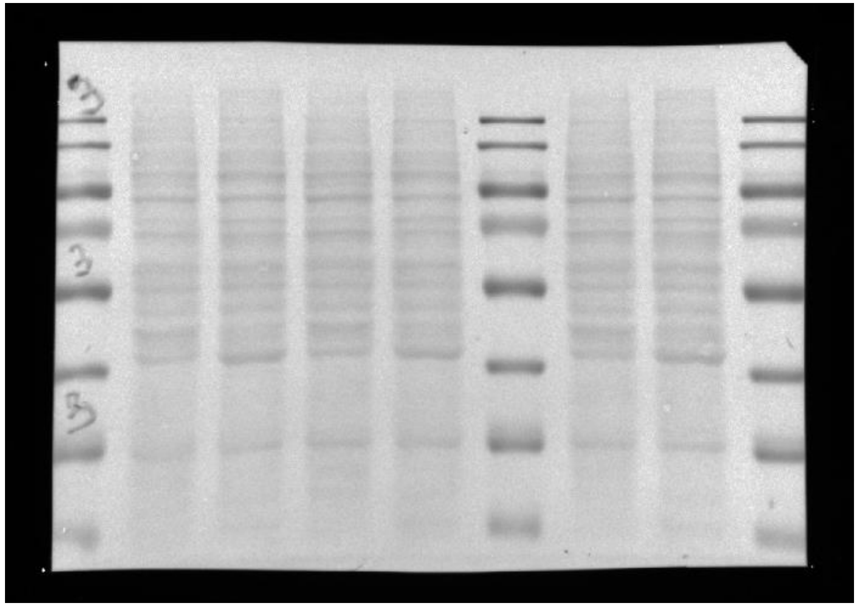

CDK6 WM9 (Fig.5.) CTRL- control R- resistant

CDK6 **Repetition 1,2,3**

CTRL R CTRL R CTRL R

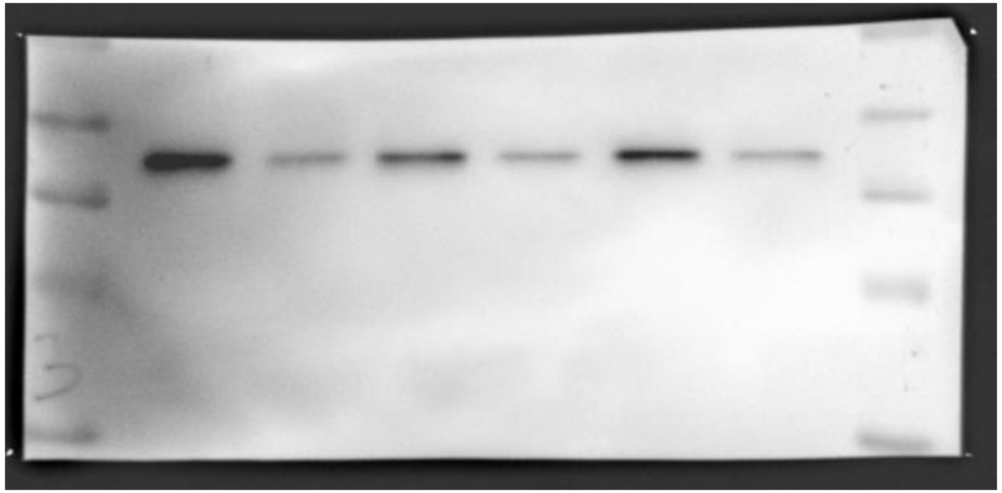

Ponceau Staining

CTRL R CTRL R CTRL R

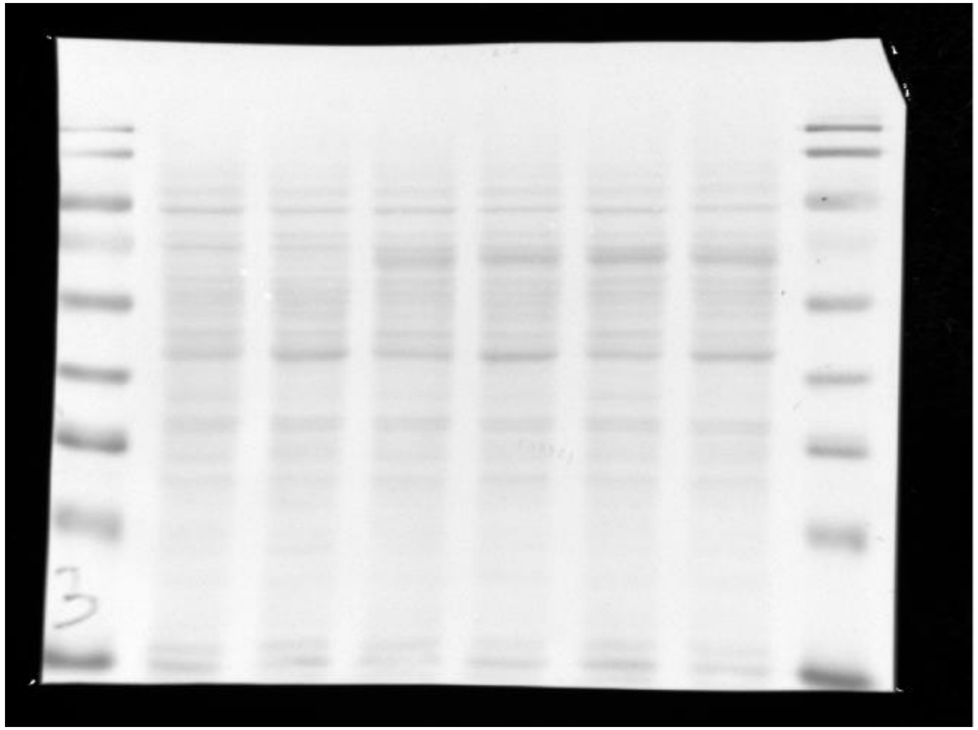

CDK6 Hs294T (Fig.5.) CTRL- control R- resistant

CDK6 **Repetition 1,2,3**

CTRL R CTRL R CTRL R

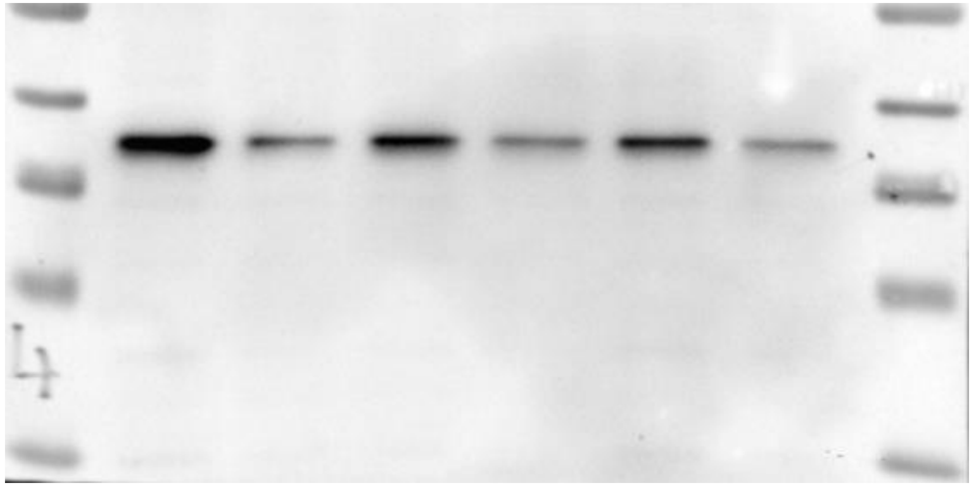

Ponceau Staining

CTRL R CTRL R CTRL R

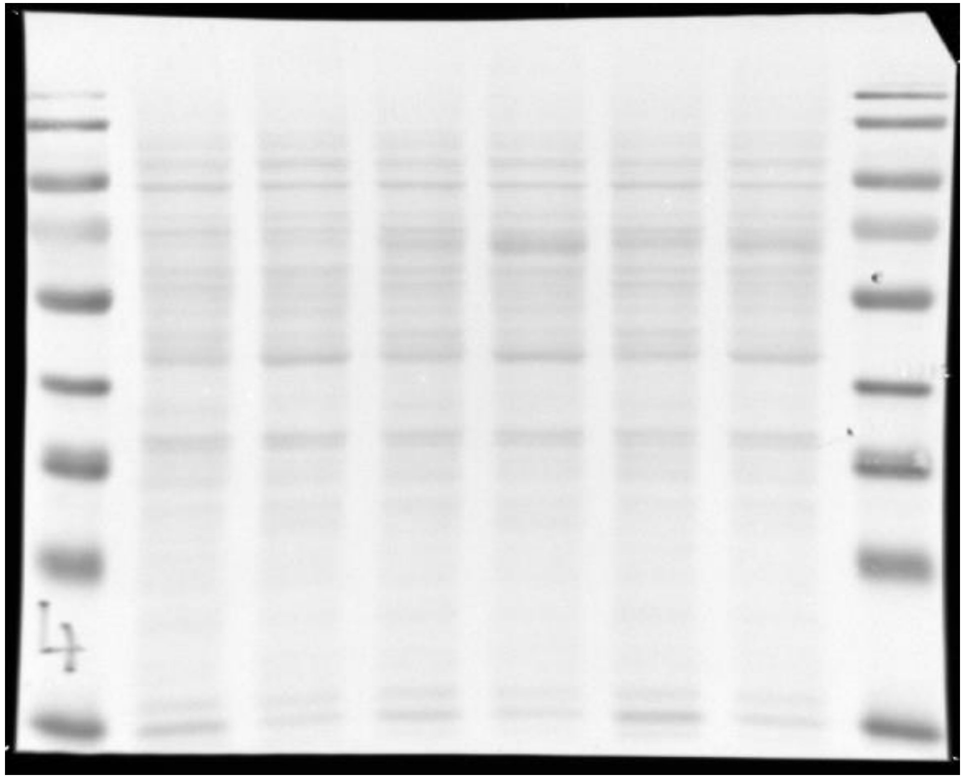

p18 WM9 (Fig.5.) CTRL- control R- resistant

p18 **Repetition 1,2,3** **Ponceau Staining**

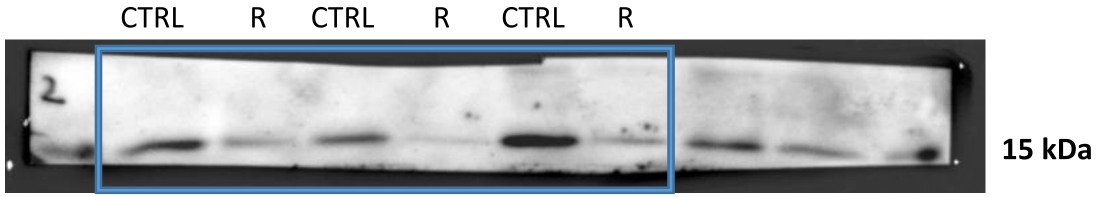

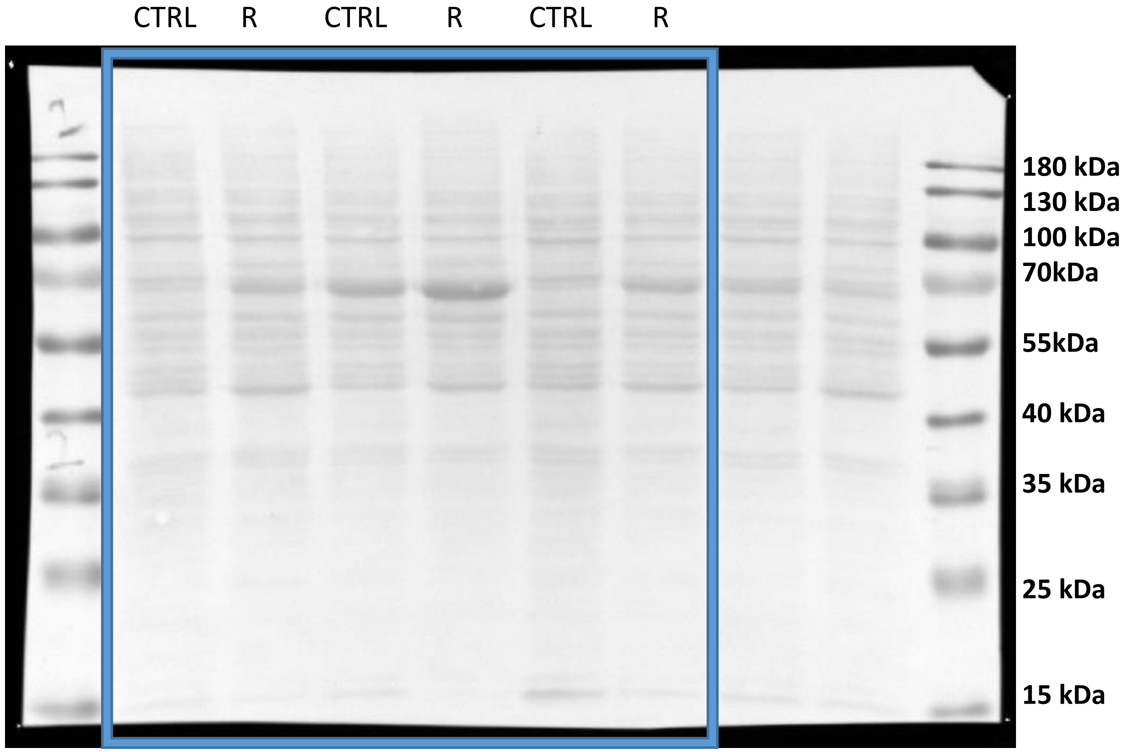

p18 Hs294T (Fig.5.) CTRL- control R- resistant

p18 **Repetition 1,2**

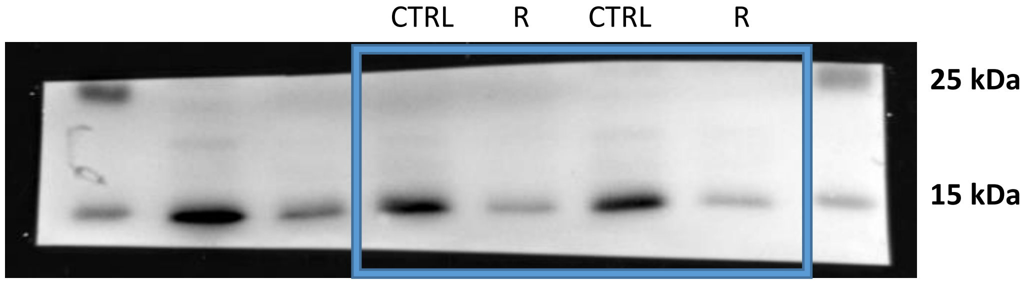

Ponceau Staining

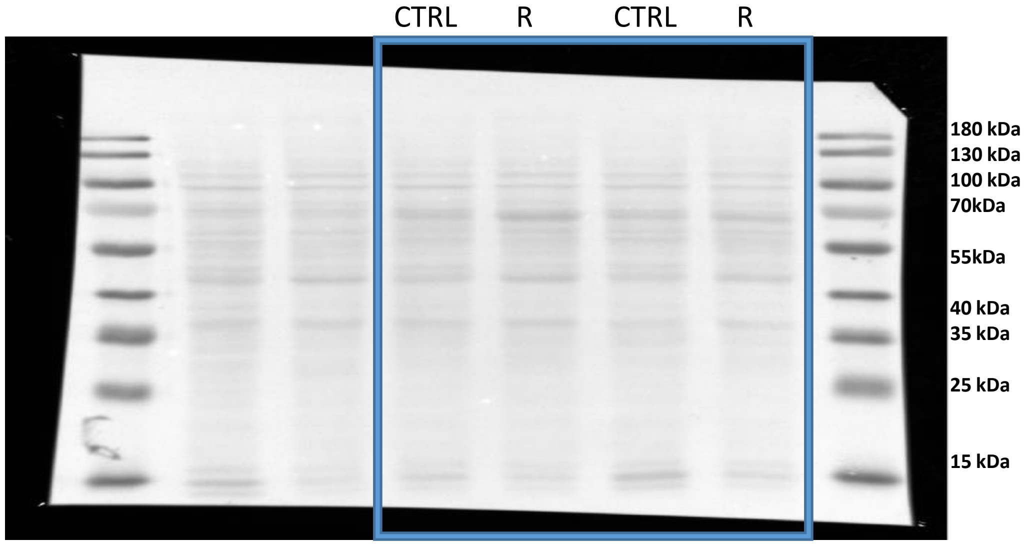

p18 **Repetition 3**

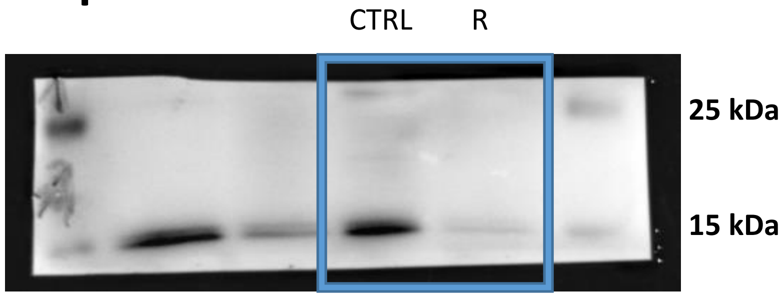

Ponceau Staining

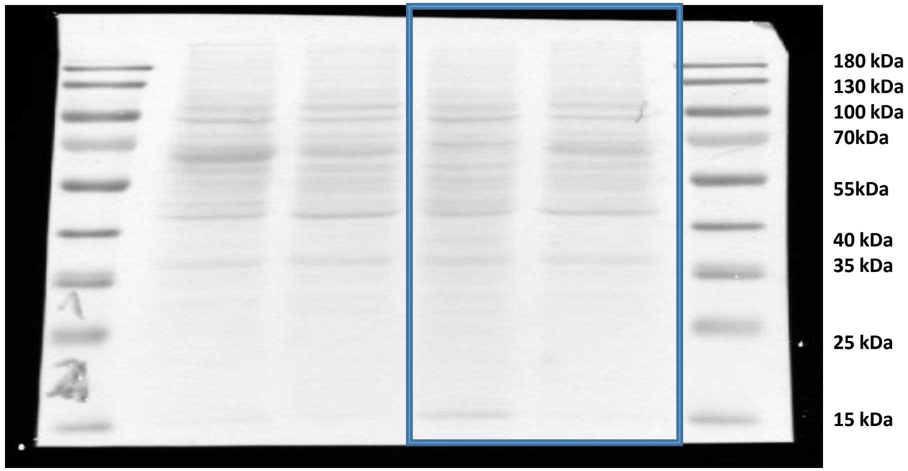

p21 WM9 (Fig.5.) CTRL- control R- resistant

p21 **Repetition 1,2,3** **Ponceau Staining**

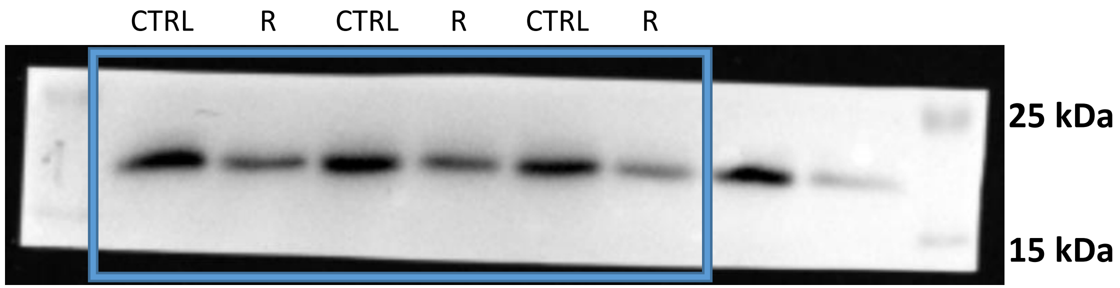

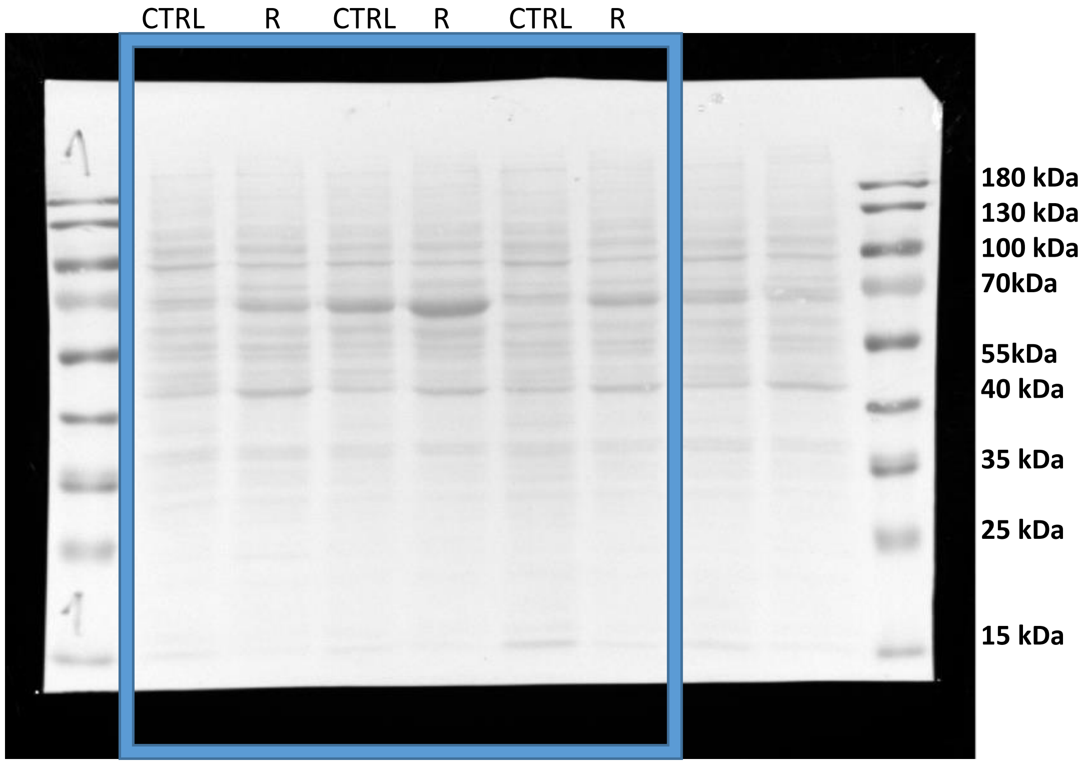

p21 Hs294T (Fig.5.) CTRL- control R- resistant

p21 **Repetition 1,2,3**

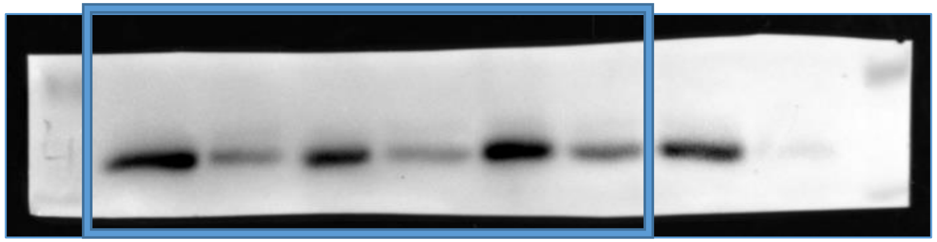

Ponceau Staining

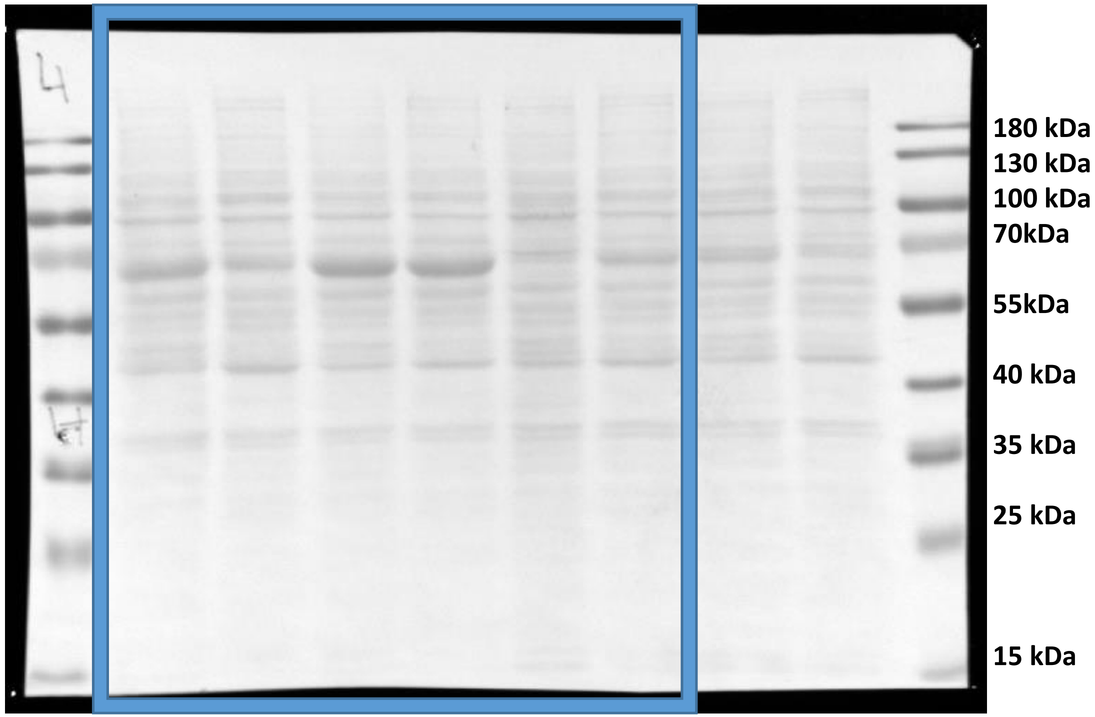

p27 WM9 (Fig.5.) CTRL- control R- resistant

p27 **Repetition 1,2,3** **Ponceau Staining**

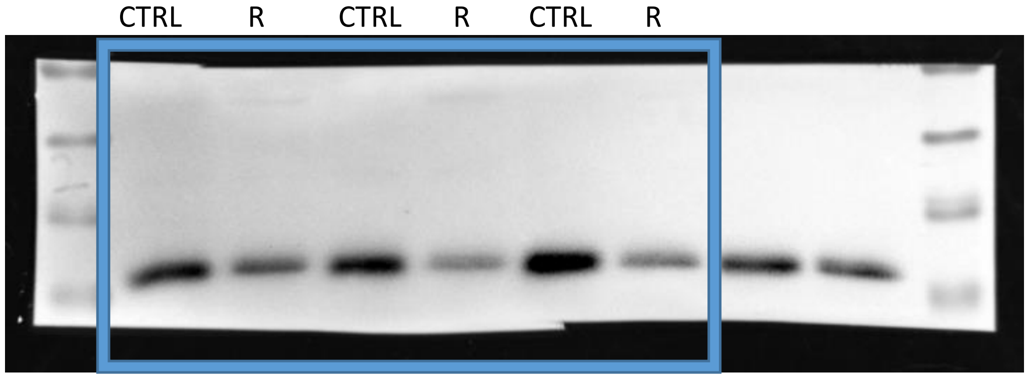

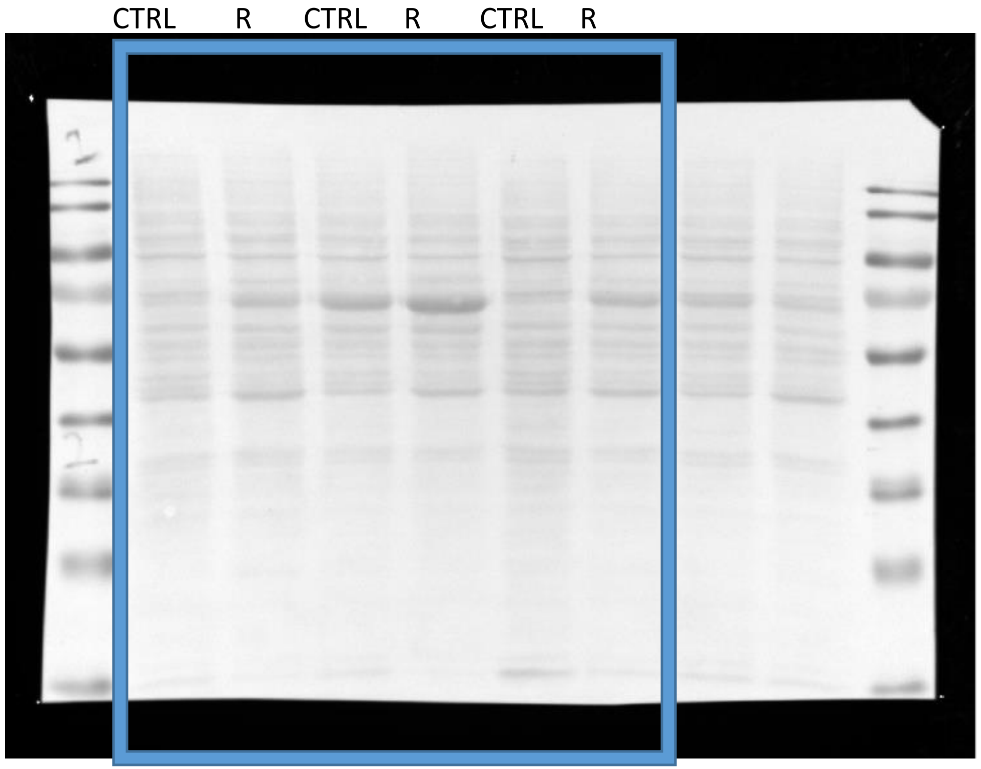

p27 Hs294T (Fig.5.) CTRL- control R- resistant

p27 **Repetition 1,2** **Ponceau Staining**

CTRL R CTRL R

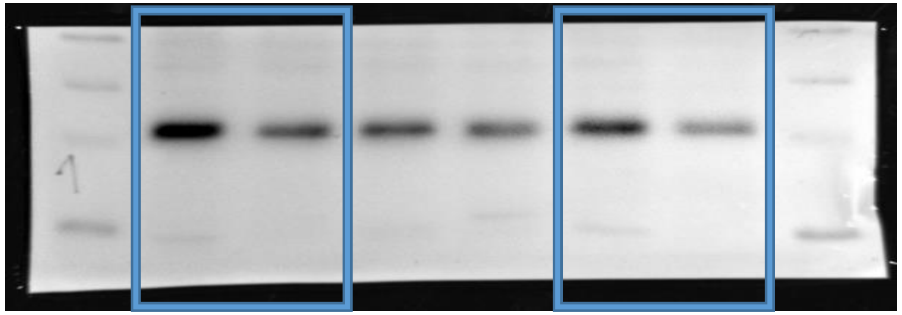

CTRL R CTRL R

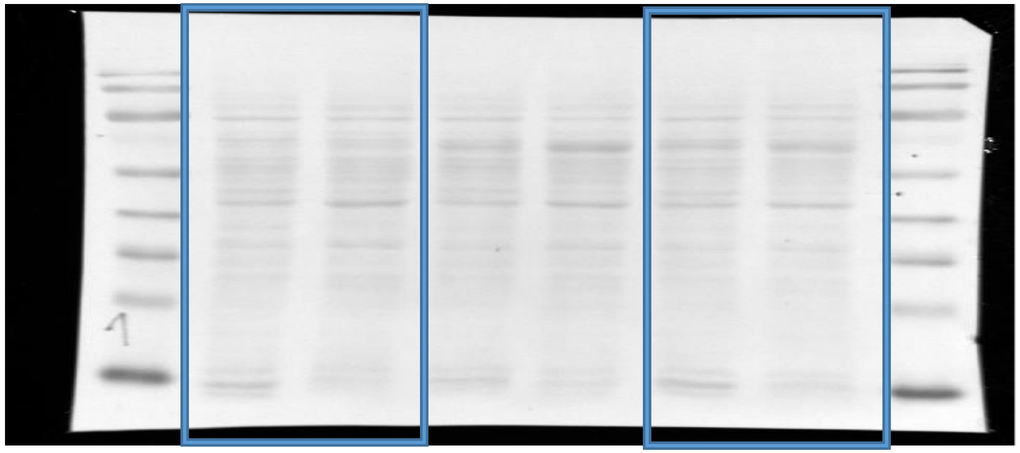

p27 **Repetition 3,4** **Ponceau Staining**

CTRL R CTRL R

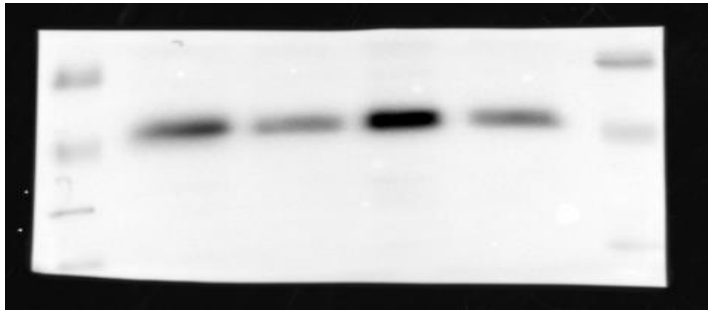

CTRL R CTRL R

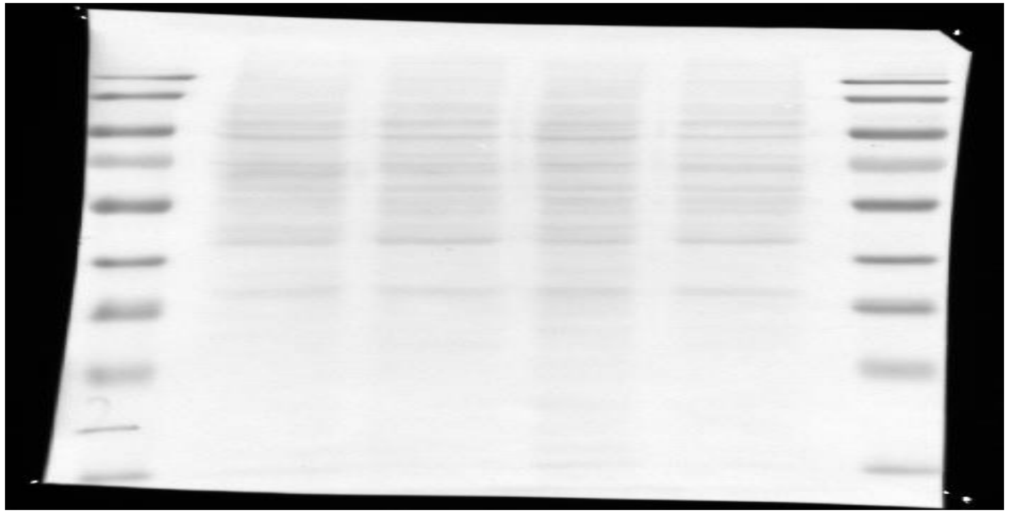

ALCAM WM9 (Fig.6.) CTRL- control R- resistant

ALCAM **Repetition 1,2** **Ponceau Staining**

CTRL R marker CTRL R

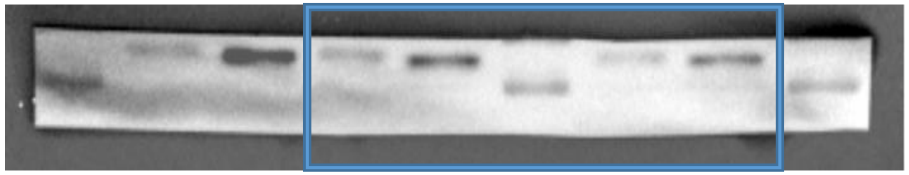

CTRL R CTRL R

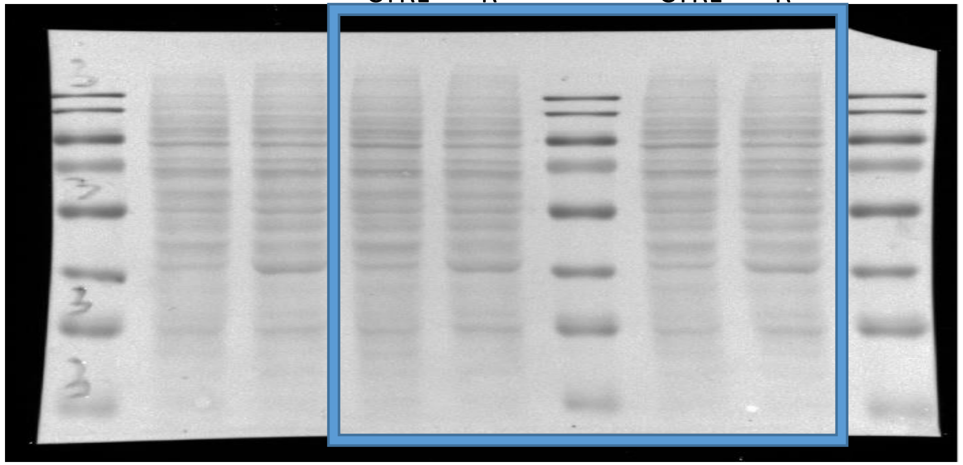

ALCAM **Repetition 3,4** **Ponceau Staining**

CTRL R CTRL R

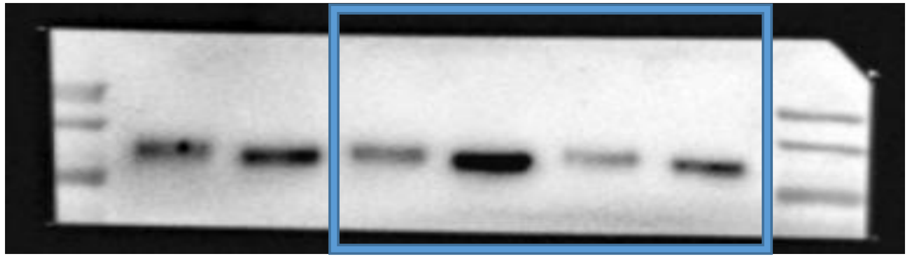

CTRL R CTRL R

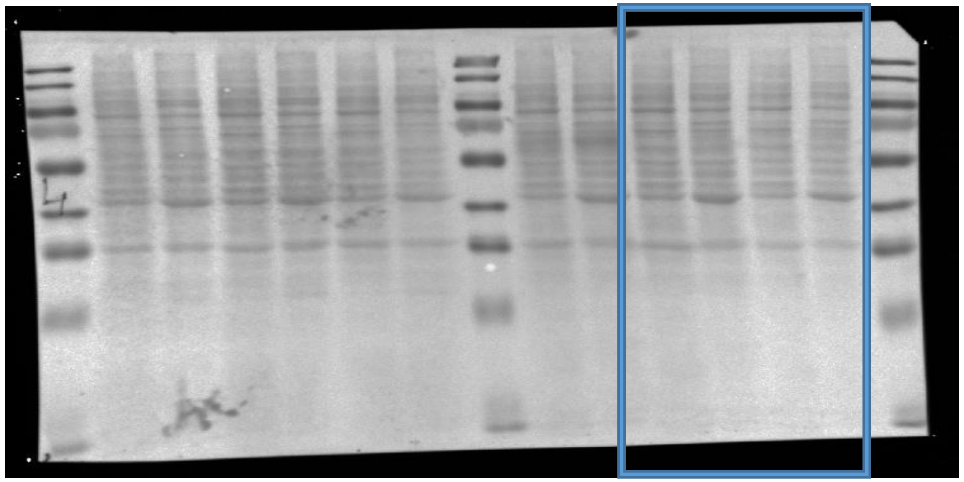

ALCAM Hs294T (Fig.6.) CTRL- control R- resistant

ALCAM **Repetition 1,2,3** **Ponceau Staining**

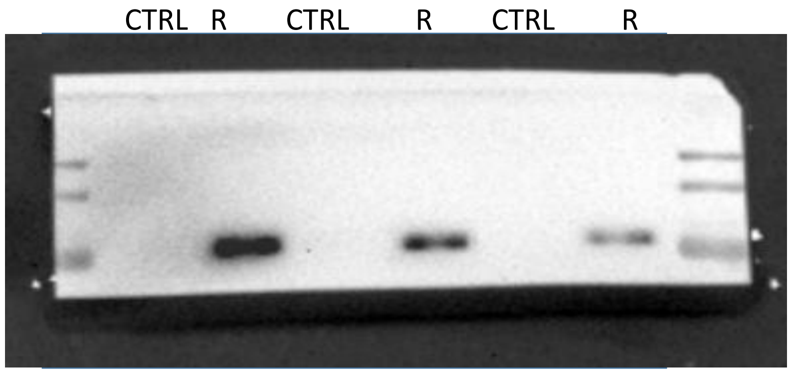

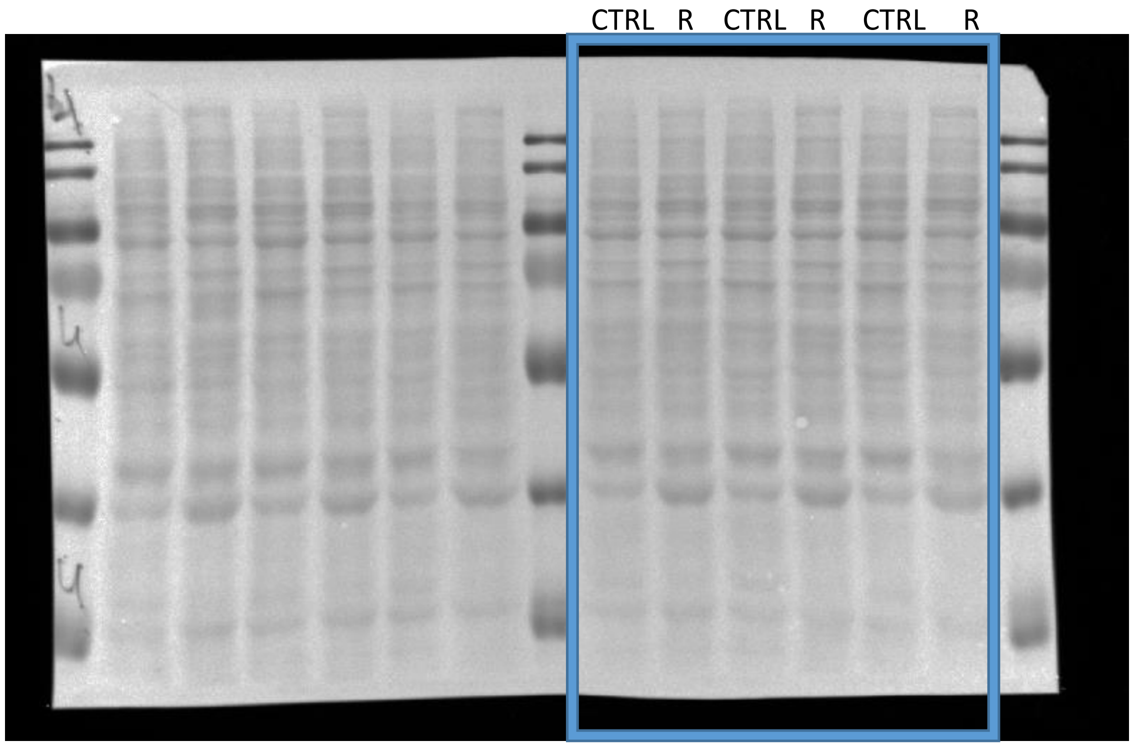

TGFβR1 WM9 (Fig.7.) CTRL- control R- resistant

TGFβR1

Repetition 1,2,3

Ponceau Staining

CTRL R CTRL R marker CTRL R

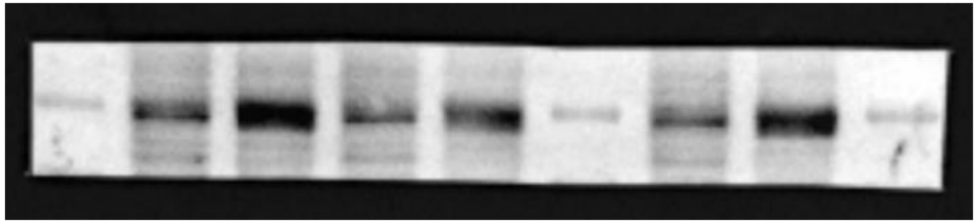

CTRL R CTRL R CTRL R

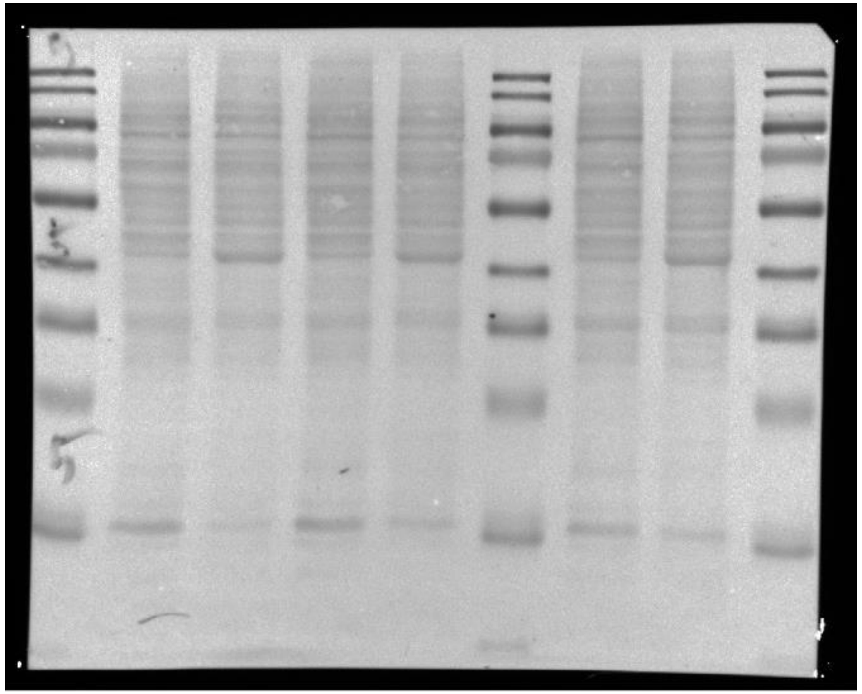

TGFβR1 Hs294T (Fig.7.) CTRL- control R- resistant

TGFβR1 **Repetition 1,2,3** **Ponceau Staining**

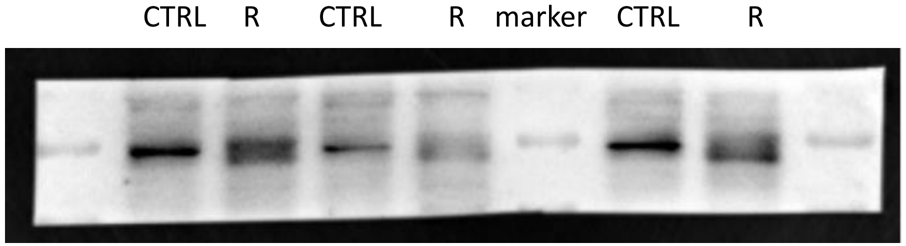

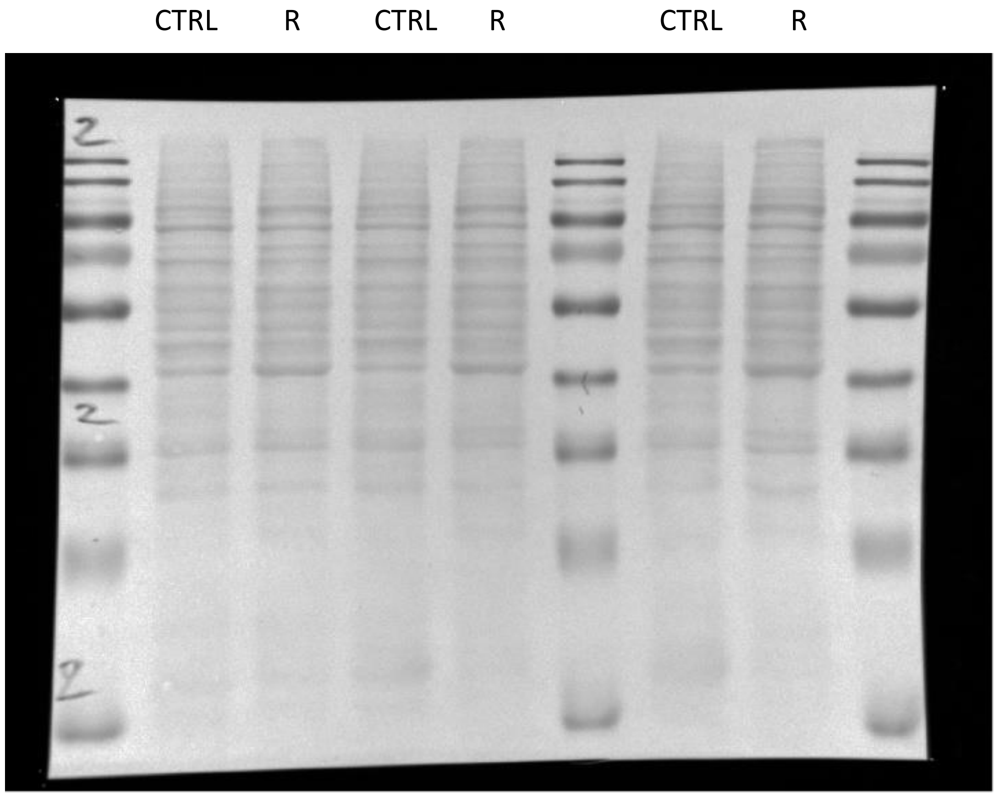

TGFβR3 WM9 (Fig.7.) CTRL- control R- resistant

TGFβR3 **Repetition 1,2** **Ponceau Staining**

CTRL R marker CTRL R

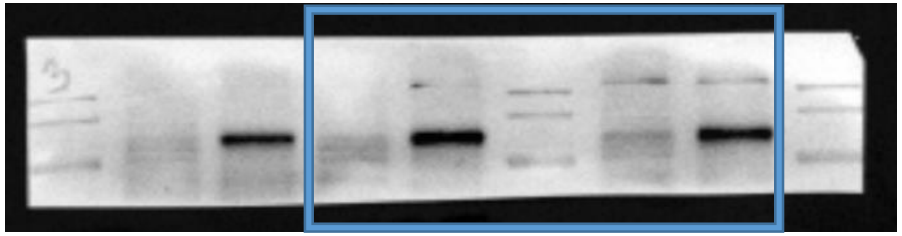

CTRL R CTRL R

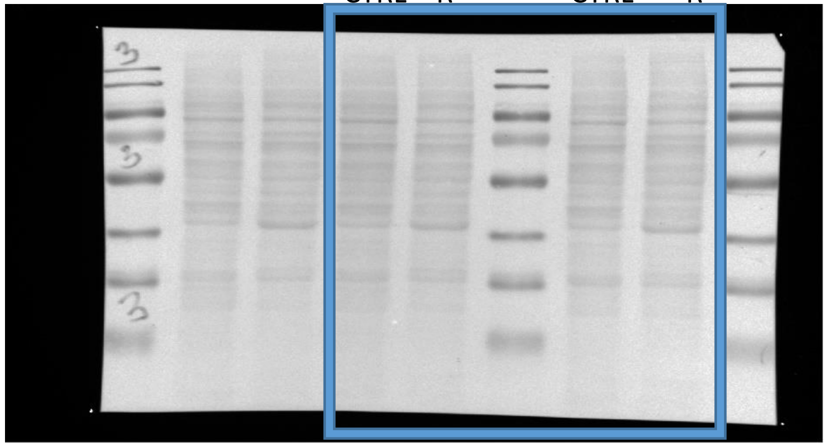

TGFβR3 **Repetition 3** **Ponceau Staining**

CTRL R

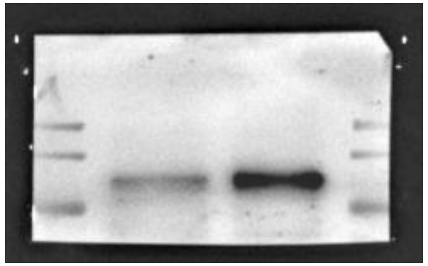

CTRL R

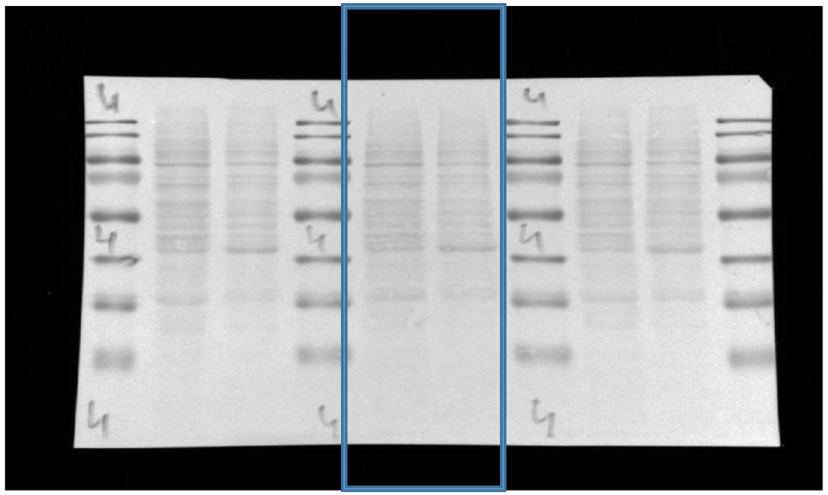

TGFβR3 Hs294T (Fig.7.) CTRL- control R- resistant

TGFβR3 **Repetition 1,2**

CTRL R CTRL R

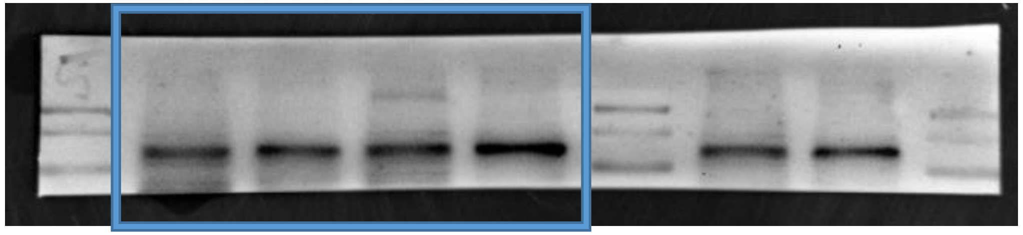

Ponceau Staining

CTRL R CTRL R

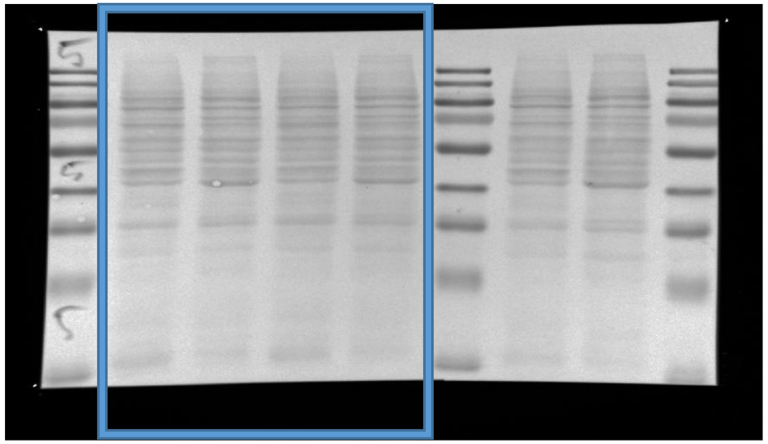

TGFβR3 **Repetition 3**

CTRL R

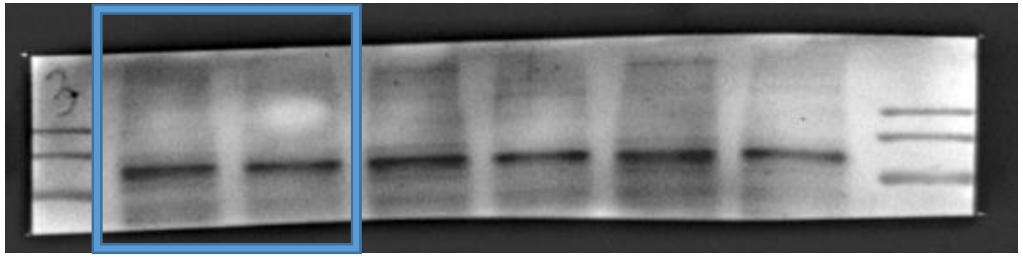

Ponceau Staining

CTRL R

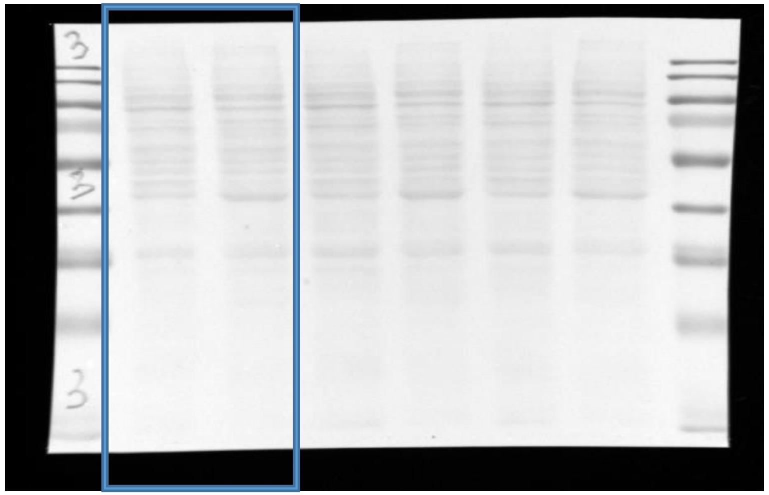

SOX2 WM9 (Fig.7.) CTRL- control R- resistant

SOX2

Repetition 1,2,3

Ponceau Staining

marker CTRL R marker CTRL R marker CTRL R marker

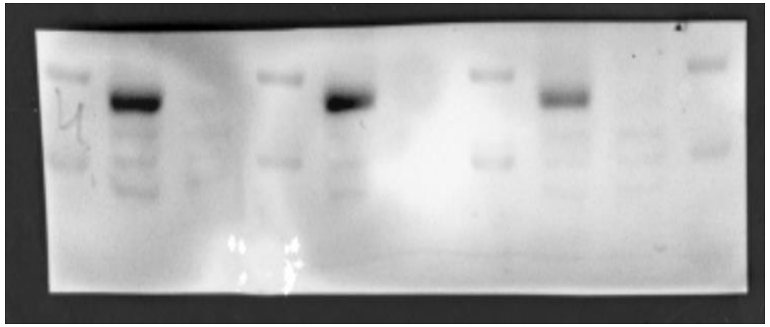

CTRL R CTRL R CTRL R

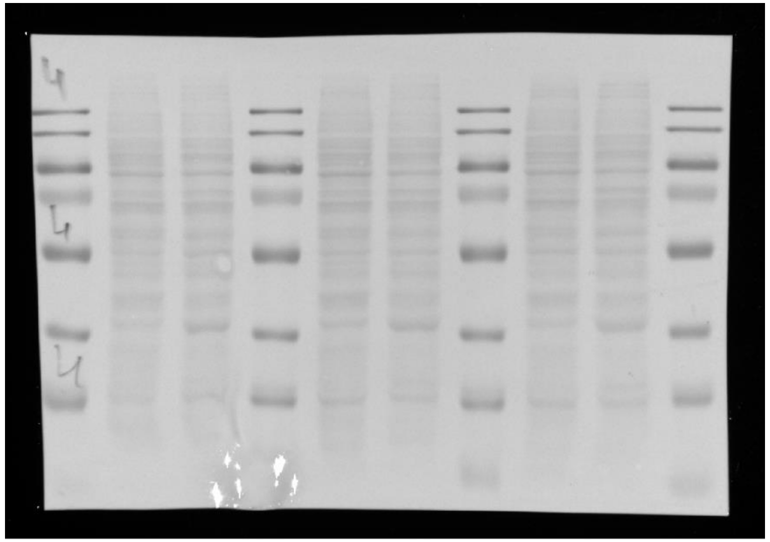

SOX2 Hs294T (Fig.7.) CTRL- control R- resistant

SOX2

Repetition 1,2,3

marker CTRL R CTRL R marker CTRL R marker

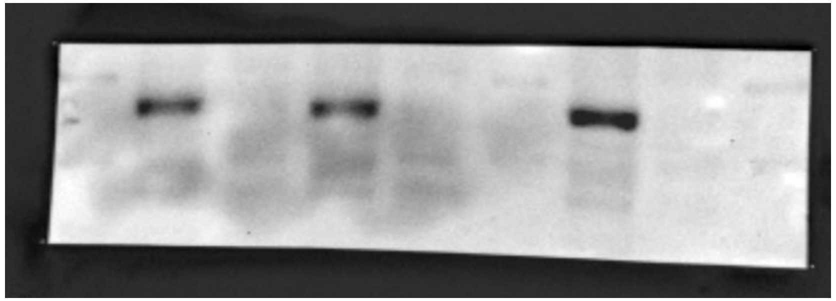

Ponceau Staining

CTRL R CTRL R CTRL R

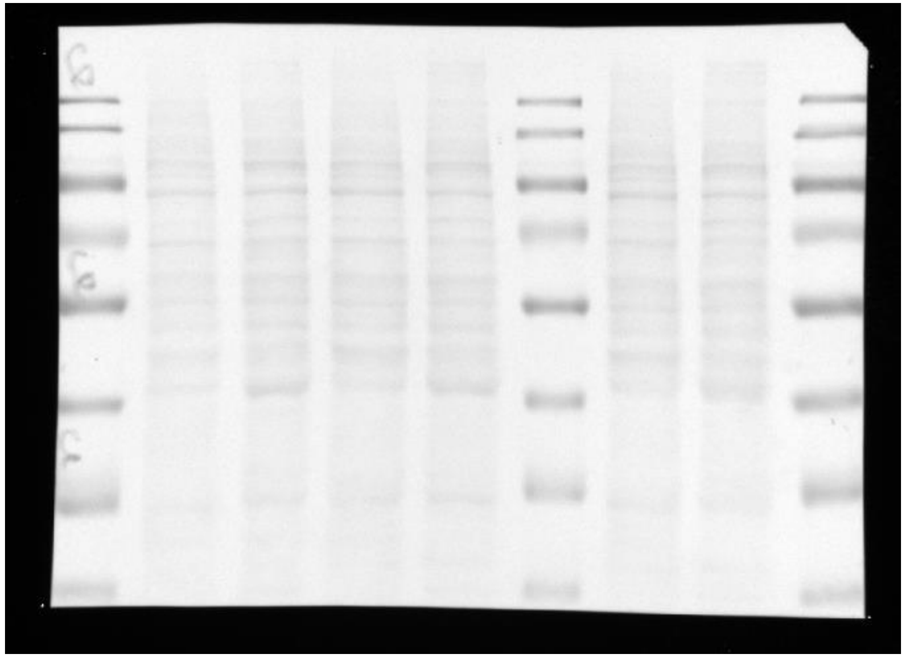

Supplement: Supplementary file 2 — Supplementary Material 2. [file 12964_2024_1788_MOESM2_ESM.pdf]
